# SARS-CoV-2-induced phosphorylation and its pharmacotherapy backed by artificial intelligence and machine learning

**DOI:** 10.2144/fsoa-2023-0112

**Published:** 2024-05-15

**Authors:** Fouzia Qamar, Zubair Sharif, Jawaria Idrees, Asif Wasim, Sana Haider, Saad Salman

**Affiliations:** 1 Department of Biology, Lahore Garrison University, Lahore-54000, Punjab, Pakistan; 2 Faculty of Medical Laboratory Sciences, Superior University, Lahore-54000, Punjab, Pakistan; 3 Khyber Pakhtunkhwa Education Monitoring Authority, Khyber-Pakhtunkhwa, Peshawar-25000, Pakistan; 4 Department of Pharmacy, CECOS University of IT & Emerging Sciences, Peshawar-25000, Khyber Pakhtunkhwa, Peshawar, Pakistan

**Keywords:** monoclonal antibodies, nucleocapsid protein, phosphorylation, proteomics, spike protein

## Abstract

**Aims:** To investigate the role of phosphorylation in SARS-CoV-2 infection, potential therapeutic targets and its harmful genetic sequences. **Materials & Methods:** Data mining techniques were employed to identify upregulated kinases responsible for proteomic changes induced by SARS-CoV-2. Spike and nucleocapsid proteins' sequences were analyzed using predictive tools, including SNAP2, MutPred2, PhD-SNP, SNPs&Go, MetaSNP, Predict-SNP and PolyPhen-2. Missense variants were identified using ensemble-based algorithms and homology/structure-based models like SIFT, PROVEAN, Predict-SNP and MutPred-2. **Results:** Eight missense variants were identified in viral sequences. Four damaging variants were found, with SNPs&Go and PolyPhen-2. Promising therapeutic candidates, including gilteritinib, pictilisib, sorafenib, RO5126766 and omipalisib, were identified. **Conclusion:** This research offers insights into SARS-CoV-2 pathogenicity, highlighting potential treatments and harmful variants in viral proteins.

## Plain language summary

This study explores the process called phosphorylation, which involves adding phosphate groups to certain proteins, influences the way the SARS-CoV-2 virus causes disease. The virus manipulates host enzymes to help it spread and survive. Researchers used data analysis techniques to identify the proteins that play a role in this process, aiming to find potential targets for treatments. They analyzed genetic sequences of key virus proteins and used various tools to predict harmful mutations. The study found several promising compounds that could be used to target the virus. Further research and experiments are needed to confirm their effectiveness as COVID-19 treatments.

## Tweetable abstract

This research explored the process called phosphorylation, which involves adding certain molecules to proteins, affects how the SARS-CoV-2 virus makes people sick. The virus uses our own cell's machinery to help it spread. Researchers used computer analysis to find out which proteins are involved in this process, hoping to find new ways to treat COVID-19. They studied the genetic code of important parts of the virus and used computer programs to predict if there were harmful changes in the code. They found some potential medicines that could be used to fight the virus and reduce its harm, but more research and testing are needed to be sure.

## Abstract (plain language summary)

Phosphorylation, involving phosphate group addition to viral and host proteins, influences SARS-CoV-2 pathogenicity.

Study uses data mining to identify upregulated kinases responsible for proteomic changes induced by SARS-CoV-2.

Spike and nucleocapsid protein sequences of SARS-CoV-2 were analyzed using predictive and structure-based tools.

The tools categorized into groups such as protein structure analysis, consensus-based, homology-based, and supervised methods.

Eight missense variants identified in viral sequences, with one potentially damaging variant.

SNPs&Go and PolyPhen-2 predicted four distinct possibly damaging variants of SARS-COV-2.

Promising therapeutic candidates include gilteritinib, pictilisib, sorafenib, RO5126766 and omipalisib.

These compounds could mitigate SARS-CoV-2 virulence and pathogenicity by modulating phosphorylation pathways and addressing viral protein variants.

The current study is not but a comprehensive analysis crucial to understanding disruptions in spike and nucleocapsid protein's structure and function.

MutPred2 uses a grouping-based machine learning model trained on positive-unlabeled data.

MutPred2 demonstrates probabilities to interpret pathogenicity and mutation scores.

Enhances understanding of genetic variations' impact on protein structure and function.

SNAP2 employs a neural network-based classifier with 848 input nodes, 25 hidden nodes and two output nodes (neutral and deleterious).

The prediction analysis cover structural, functional, secondary structure, localization, transmembrane domains, catalytic functions, interactions, post-translational modifications, metal-binding and allosteric properties.

Drugs and kinase inhibitors mapped to specific phosphorylation sites.

Kinases and phosphatases are phospho-transferases, regulating cellular protein form and function.

Human genome contains approximately 156 phosphatases and 568 protein kinases, involved in biological regulation.

Dysregulation of kinases and phosphatases can have serious health consequences.

Mutations in these enzymes can lead to disorders, and inhibitors are used as pharmacological tools.

Protein Kinase Inhibitors (PKIs) and Phosphatase Inhibitors (PIs) are in experimental and clinical development, with various attributes and applications.

The use of multiple methods increases prediction robustness in the research.

Further investigation and experimental validation required to assess their efficacy as COVID-19 treatments.

## Graphical abstract

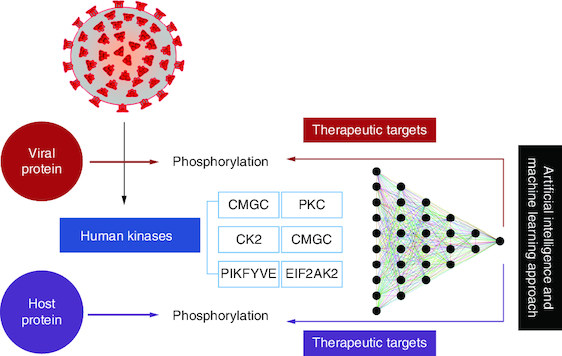

SARS-CoV-2, a member of the coronavirus family, has unleashed a global pandemic with far-reaching consequences [1,2]. The relentless emergence of novel variants has cast a shadow over the pace of COVID-19's evolution, perpetuating its impact. Beyond its disruptive effects on host cells, SARS-CoV-2 exerts a profound influence on the intricate internal machinery of the cell through post-translational modifications, most notably phosphorylation. Research has unveiled that SARS-CoV-2 infection serves as a potent trigger for the substantial phosphorylation of both host and viral proteins, implicating a complex web of molecular events [3,4]. Concurrently, the infection orchestrates the modulation of diverse host signaling pathways, a testament to the multifaceted interactions at play. Notably, this interplay unveils a striking interconnectivity between seemingly disparate phosphoproteins and their interactome. Furthermore, certain differentiation proteins and kinases have emerged as pivotal targets for potential COVID-19 therapeutics, shedding light on avenues for intervention [5]. Disruptions in the delicate balance of protein kinases and phosphatases regulation have been shown to carry profound implications for health, as elucidated in the comprehensive Supplementary Table 1, which provides a self-explanatory account of these deleterious consequences.

Numerous kinases and protein entities have emerged as promising and prospective targets for therapeutic interventions against COVID-19. To elucidate the intricate landscape of global phosphorylation changes induced by SARS-CoV-2 infection, a comprehensive analysis has been undertaken [^4-6^]. This analysis encompasses a diverse array of cell lines, including A549-ACE2, iAT2s (induced pluripotent stem cell-derived alveolar type 2 cells), CaCo-2 (human colon cancer cell line) and Vero E6 (chimpanzee kidney epithelial cell line). Such cellular models have been strategically chosen to provide insights into the dynamic alterations in phosphorylation patterns across various tissue types and to comprehensively investigate the molecular events associated with SARS-CoV-2 infection [^6-9^].

Host factors intricately orchestrate the expression of approximately 27 proteins encoded within the SARS-CoV-2 genome, encompassing four structural, 15-nonstructural and eight auxiliary proteins. Exemplary instances of structural proteins include spike (S), envelope (E), nucleocapsid (N) and membrane (M), numbering three, five, nine and ten, respectively [10]. Notably, the interplay between host and viral components undergoes significant shifts during the course of SARS-CoV-2 infection. A noteworthy trend emerges with a decrease in host proteins, concomitant with a substantial augmentation in the abundance of viral proteins, underscoring the virus's ability to modulate cellular protein expression to its advantage [^11-13^]. Of particular interest is the pervasive phosphorylation of both viral and host proteins subsequent to their translation, an event that exhibits a marked escalation in response to SARS-CoV-2 infection. This phosphorylation cascade, orchestrated by the virus, exerts control over multiple signaling pathways. Noteworthy among the pathways impacted are protein trafficking, translation and RNA processing, all of which are subject to intricate redesign by SARS-CoV-2. Consequently, this molecular reprogramming places cells under duress, disrupting their normal life cycles and impeding their physiological functions. This molecular hijacking is particularly pronounced in the respiratory system, where SARS-CoV-2 exerts a profound influence, ultimately subverting host cellular processes [6,9,14].

Phospho-proteomics investigations have emerged as invaluable tools, enabling researchers to delve into the intricate molecular landscapes underpinning signaling cascades, host–pathogen interactions, pathophysiological mechanisms and the ramifications of SARS-CoV-2 infection [7,11,15]. Notably, these analyses have unveiled a wealth of information, with approximately 70 phosphorylation sites identified within the viral N and S proteins alone. Furthermore, the comprehensive cataloging of more than 15,000 phosphorylation sites [3,5,11] has been elucidated, spanning a vast array of host phosphoproteins intricately interwoven within numerous signaling cascades.

The dynamic alterations within phosphoproteins and their associated phosphorylation sites serve as a promising avenue for the investigation of efficacious therapeutic strategies against COVID-19 [12], with particular emphasis on the development of potent kinase inhibitors. SARS-CoV-2 exerts a profound influence on a diverse cadre of kinases, including, but not limited to, PKC, CK2, CMGC and CDK, thereby orchestrating the regulation of pivotal signaling pathways, such as EGFR, AKT, GFR signaling, the MAPK cascade, autophagy, TGF and interferon pathways. Promising antiviral activities have been demonstrated in clinical trials for several US FDA-approved AXL inhibitors, namely gilteritinib and bencentinib, along with p38 inhibitors such as ARRY-797, ralimetinib, SB203580 and MAPK13-IN-1. The CK2 inhibitor silmitasertib, the PIKFYVE inhibitor apilimod, and the CDK inhibitor dinaciclib have all exhibited potential against SARS-CoV-2. Notably, certain drugs, including gilteritinib, silmitasertib and ARRY-797, initially developed for alternative therapeutic indications, have now been strategically repurposed to combat the COVID-19 pandemic [^8-15^]. These findings underscore the multifaceted molecular landscape underlying SARS-CoV-2 infection and illuminate promising avenues for therapeutic intervention [16].

Antisense oligonucleotides (ASOs) constitute a class of short, single-stranded DNA sequences that exert their biological effects by binding to complementary mRNA sequences, thereby instigating mRNA cleavage [9,12,17]. This precise molecular interaction serves as a cornerstone for the selective degradation of target mRNA molecules, underscoring its pivotal role in ameliorating genetic anomalies. ASOs hold immense therapeutic promise as potential pharmacological agents, adept at rectifying genetic disorders. This modality hinges on the deployment of single-stranded oligonucleotides, which can elicit their therapeutic effects either through the catalytic degradation of the target RNA or by obstructing the binding of critical cellular factors [^18-20^]. The potency of antisense technology lies in its capacity to modulate gene expression, thereby rendering it a versatile tool for mitigating a broad spectrum of diseases, including but not limited to acquired immune deficiency syndrome, influenza and various forms of cancer. Notably, in the context of the ongoing global pandemic, antisense technology emerges as a particularly promising approach for addressing infections caused by coronaviruses, with a specific focus on combating the pathogenicity of COVID-19 [9,^15-21^].

Herein, we report a comprehensive analysis of potentially pathogenic sequences within the spike and nucleocapsid proteins of SARS-CoV-2 across diverse strains prevalent in the Pakistani population. Leveraging cutting-edge artificial intelligence (AI) and machine learning (ML) techniques, we have employed advanced computational methodologies to identify deleterious genetic variations within these viral proteins. Furthermore, we have conducted an exhaustive review of the existing scientific literature to explore potential therapeutic avenues. Our analysis incorporates proteomic profiling, with a focus on kinase-targeting strategies, and explores the feasibility of antisense technology as a prospective treatment modality. The amalgamation of these computational and literature-based approaches provides a robust foundation for advancing our understanding of the molecular underpinnings of SARS-CoV-2 pathogenicity and the development of targeted therapeutic interventions.

We have utilized a combination of data mining techniques, predictive analysis and multiple computational tools, including both supervised-based and structure-based methods, to explore the impact of phosphorylation and identified novel potential therapeutic targets in the context of SARS-CoV-2. This multipronged approach sets our study apart from previous research. We utilized extensive datasets that are employed in our analysis. Utilizing 60 sequences for the spike protein and 100 sequences for the nucleocapsid protein of SARS-CoV-2 demonstrate the depth and comprehensiveness of the current investigation. This dataset can be utilized for future research studies due to its uniqueness and significance and adds to the current understanding of the virus as well as its therapeutics. Our study emphasized the diversity of predictive tools for such studies. By combining both supervision-based and structure-based methods, we provided a holistic view of potential damaging variants within the viral sequences. This combination of tools is crucial for a comprehensive analysis and differs from previous studies that may have focused on only one or two types of tools.

## Materials & methods

### Machine learning approach

#### Protein sequence retrieval

In this section, we outline the methodology employed for the retrieval of the SPIKE protein sequences of SARS-CoV-2 in FASTA format. The protein sequences were obtained from the National Center for Biotechnology Information (NCBI) database, accessible at https://www.ncbi.nlm.nih.gov/protein/. This process of sequence retrieval is a fundamental step in our research, providing the essential raw data required for subsequent analyses and investigations [^21-23^].

### Identification of deleterious missense single-nucleotide polymorphisms (SNPs) in the Spike protein

The objective of this section is to systematically identify and characterize missense SNPs within the spike protein of SARS-CoV-2 that exerts a detrimental influence on protein structure and function. This task involves the utilization of various bioinformatics tools, each employing distinct algorithms and methodologies. The selected tools are categorized into four distinct groups based on their underlying algorithms and approaches. These categories encompass methods rooted in protein structure and function analysis, consensus-based methods, homology-based techniques and supervised methods. By leveraging these computational instruments, we aim to predict the functional consequences of mutations within the protein sequences, ultimately discerning the most damaging phenotypic alterations. This comprehensive analysis serves as a critical step in our study to unveil potential disruptions in the spike protein's structure and function, shedding light on their implications for viral behavior and pathogenesis.

#### Sequence-based homology method for missense SNP assessment

In this section, we delve into the sequence-based homology method, a vital approach employed for the classification of non-synonymous single-nucleotide polymorphisms (nsSNPs). The primary objective of this methodology is to discern nsSNPs that carry significant phenotypic consequences, particularly those associated with diseases or those that have a deleterious impact on protein function [^24-28^].

##### SIFT: sorting intolerant from tolerant

SIFT, an acronym for ‘sorting intolerant from tolerant’, was introduced in 2001 as a pioneering bioinformatics tool designed to predict the effect of coding variants on protein function. Since its inception, SIFT has become a cornerstone in the field of missense variation analysis, renowned for its reliability and widespread adoption. Its core purpose revolves around estimating the probability that a substitution of amino acids will exert a detrimental effect on protein function. To accomplish this, SIFT leverages the concept of sequence homology, capitalizing on the understanding that evolutionarily conserved regions within proteins are less tolerant to mutations. Consequently, substitutions, insertions, deletions or any alterations occurring within these conserved regions are more likely to disrupt the protein's functional role.

The SIFT algorithm initiates its analysis by utilizing a query protein, which is systematically compared against a comprehensive protein database to identify homologous protein sequences. Subsequently, SIFT evaluates the composition of amino acids within these homologous sequences and calculates a specific score to gauge the likelihood of a substitution's impact on protein function [29].

SIFT employs a multifaceted methodology, involving the identification of closely related sequences, multiple sequence alignment, estimation of alignment-based replacement probabilities, and the determination of a tolerance index for the amino acid substitutions. The culmination of these steps yields a SIFT score, which is normalized and ranges from 0 to 1. Importantly, a score of 0.05 serves as the critical cutoff value in SIFT analysis. Variations with scores falling between 0 and 0.05 are anticipated to have a significant impact on protein function, rendering them harmful [^29-31^].

The comprehensive functionality of the SIFT tool is instrumental in our research for evaluating missense SNPs in the spike protein of SARS-CoV-2. It serves as a pivotal component in our endeavor to assess the potential functional consequences of genetic variations in this critical viral protein. For additional details and access to the tool, please refer to https://sift.bii.a-star.edu.sg/.

##### PROVEAN: protein variation effect analyzer

PROVEAN, short for protein variation effect analyzer, is a powerful bioinformatics tool integral to our study. Its primary function is to predict the functional impact of alterations within protein sequences, encompassing minor insertions or deletions as well as single amino acid substitutions. This prediction is made by comparing the query protein sequence to a comprehensive database of associated protein sequences. PROVEAN plays a critical role in identifying variants that are linked to diseases or that have a detrimental effect on protein function.

PROVEAN distinguishes itself through its ability to efficiently detect a wide range of variations, including single or multiple amino acid substitutions, insertions, and deletions. Notably, it offers a versatile analysis at the proteomic level, making it a valuable asset for our research.

The PROVEAN tool provides three main methods for identifying harmful variants: PROVEAN Protein, PROVEAN Protein Batch, and PROVEAN Genome Variants. While PROVEAN Protein is suitable for analyzing variants in any organism, the latter two methods are specifically tailored for humans. To calculate the final PROVEAN score, the tool employs an average score calculation process that involves clustering BLAST hits using a 75% global sequence identity parameter. Subsequently, the prediction is generated based on the first 30 clusters of closely related sequences, forming the supporting sequence collection. A threshold score of -2.5 is applied, signifying that a protein variant is expected to have a deleterious effect if the PROVEAN score falls at or below this predefined threshold. Conversely, variants are deemed to have a ‘neutral’ effect if their PROVEAN score exceeds -2.5 [^28-30^].

PROVEAN's sophisticated capabilities are pivotal in our research to assess the impact of missense SNPs within the spike protein of SARS-CoV-2. It enables us to discern the potential functional consequences of genetic variations, aiding in the identification of variants with deleterious effects on protein function.

#### Methods based on supervised learning

In this subsection, we introduce methods based on supervised learning, employing advanced algorithms such as neural networks, random forests and support vector machines to predict the deleteriousness of amino acid variations. We utilized four tools in this category: SNPs&GO, MutPred, PhD-SNP and SNAP-2, to further screen and evaluate genetic variants.

##### SNPs&GO

SNPs&GO is a bioinformatics web browser that harnesses a comprehensive system, integrating data derived from protein sequences, 3D structures, protein classification profiles and protein function. This tool leverages an extensive collection of human SNPs available in various databases, enabling the correlation of protein sequences with potential deleterious effects. SNPs&GO employs multiple sources of information, including data retrieved from the Gene Ontology annotation, to predict whether a specific variant is likely to have a deleterious or neutral impact on the protein. The prediction is conducted through a GO-incorporated classifier, rigorously trained with a cross-validation strategy. SNPs&GO has demonstrated remarkable efficacy, achieving an impressive scoring accuracy of 82% for the prediction of single-point mutations in protein sequences associated with disease conditions in humans.

##### PhD-SNP

PhD-SNP utilizes a support vector machine (SVM)-based classifier with a defined cut-off value set at 0.5. The feature vector used by PhD-SNP consists of 40 characteristics, with the initial 20 residue types explicitly denoting the difference between the wild-type amino acids and the mutant amino acids added by assigning a value of -1 to the former and 1 to the latter. The remaining components are maintained at a value of 0. This unique approach has proven effective in predicting the pathogenicity of amino acid substitutions, providing valuable insights into potential deleterious effects [^21-25^].

##### MutPred2

MutPred2 employs a grouping-based model built upon recent advances in machine learning techniques, training on positive-unlabeled data. It incorporates assessments of prior probabilities and posterior probabilities, enabling the interpretation of pathogenicity and mutation scores. MutPred2 offers a comprehensive suite of predictions, encompassing structural and functional properties, secondary structure, peptide localization, transmembrane domains, protein catalytic functions, macromolecular interactions, post-translational modifications, metal-binding properties and allosteric properties. This breadth of predictions enhances our understanding of the potential impacts of genetic variations on protein structure and function [25].

##### SNAP2

SNAP2 employs a neural network-based classifier consisting of 848 nodes in the input layer, 25 nodes in the hidden layer and two nodes (one for neutral and one for deleterious) in the output layer. In each training step, examples are presented to the network and association weights are adjusted through a back propagation algorithm. SNAP2 incorporates ten different models created during tenfold cross-validation, each using distinct subsets for training and development. The final score is computed as the difference between the average score for effect and the average score for neutral, resulting in a score range from 0 (low reliability) to 9 (high reliability).

These supervised learning methods are pivotal in our research, as they provide comprehensive assessments of the potential deleteriousness of amino acid variations, aiding in the identification of variants with functional consequences. The use of multiple methods enhances the robustness of our predictions.

#### Consensus & structure-based methods

Consensus and structure-based methods form another critical category of analysis tools. While traditionally applied separately, we have chosen to use consensus-based tools, including Condel and PON-P2, alongside structure-based tools like SDM, Fold-X.

## Pharmacological modulators: mapping kinase activities

We conducted an extensive virtual screening process, employing a diverse range of theoretical and experimental methodologies, to identify potential drugs targeting various facets of SARS-CoV-2 based on insights from prior research [^9-19^]. These selected drugs, along with kinase inhibitors, were systematically mapped to specific phosphorylation sites, with the aim of discovering effective treatments for SARS-CoV-2 infection, utilizing the most recent data available [4,^20-22^]. To assess their potential, all identified drugs were compared with previously reported drug targets.

In total, we identified 87 drug candidates, among which ten had received FDA approval, 53 were undergoing clinical testing and 24 were in the preclinical development stage, as per previously documented drugs. An alternative approach involved inhibiting GSK-3 in the nucleocapsid protein [^21-25^]. Our hypothesis posited that evaluating compounds with both overlapping and distinct targets would facilitate the identification of the most pivotal molecular targets for combating SARS-CoV-2. Furthermore, we subjected 68 different drugs and substances to rigorous testing for cellular toxicity and antiviral activity at two separate research institutions, employing methods such as qRT-PCR, anti-NP antibodies, plaque assays and TCID_50_, using A549-ACE2 and Vero-E6 cell lines, all of which were included in the comprehensive analysis [4].

Kinases and phosphatases are enzymes classified as phospho-transferases, and they facilitate distinct enzymatic reactions critical for the regulation of cellular proteins' conformation and functionality [^23-28^]. These enzymes are often referred to as key orchestrators of biological processes, serving as molecular switches that influence the structural and functional aspects of cellular biomolecules [^24-30^]. Phosphorylation and dephosphorylation events play a pivotal role in modulating the activation or deactivation of a multitude of receptors and enzymes during various cellular and biological processes. The human genome encompasses approximately 156 phosphatases and 568 protein kinases, which are well-documented participants in the management and regulation of biological systems.

It is an established empirical fact, substantiated by scientific research, that the dysregulation or anomalous control of protein kinases and phosphatases can give rise to significant health ramifications, as elucidated in Supplementary Table 1 [25]. Anomalous phosphorylation events initiated by mutations within specific protein kinases and phosphatases can lead to diverse pathologies, and their mitigation can be achieved through the utilization of kinase and phosphatase inhibitors acting as pharmacological tools [26]. A multitude of protein kinase inhibitors (PKIs) and phosphatase inhibitors (PIs), endowed with diverse attributes and applications, have progressed into the realms of experimental investigation and clinical development [25,^27-31^]. The declaration of Helsinki principles were followed in our research.

## Results

### Interactions & mutations of the Spike protein of SARS-CoV-2

SARS-COV-2 interactions with other proteins are also analyzed using the STRING database. The STRING database is a well-known and widely used bioinformatics resource for the analysis of protein–protein interactions (PPIs) and functional associations among proteins. The name ‘STRING’ stands for ‘search tool for the retrieval of interacting genes/proteins.’ STRING provides a comprehensive collection of known and predicted protein–protein interactions, drawing data from various sources, including experimental data, computational predictions and text mining. Users can analyze protein interaction networks to discover enriched biological functions, pathways, and processes associated with a set of proteins (https://string-db.org/). STRING covers a wide range of organisms, from bacteria and fungi to plants and animals, making it versatile for researchers studying different species. STRING offers visualization tools to display protein interaction networks in an intuitive manner, helping researchers interpret the data effectively. Users can customize interaction confidence thresholds, select specific data sources and tailor their analyses to suit their research needs. In addition to experimentally validated interactions, STRING includes predicted interactions based on various computational algorithms, which can be useful for generating hypotheses. The database allows users to cluster proteins into functional modules or complexes within the interaction network. STRING provides information on protein functional annotations, including Gene Ontology (GO) terms and KEGG pathways [5].

A meticulous analysis was undertaken involving the selection of sixty distinct sequences of the spike protein of SARS-CoV-2, all of which were sourced from the Pakistani context. These sequences, meticulously curated by the National Center for Biotechnology Information (NCBI), underwent a comprehensive examination through multiple sequence alignment. This rigorous process aimed to discern the precise locations of mutations within these sequences. From this cohort of sixty sequences, we successfully identified eight distinct mutation points, as detailed in Table 1. To delve deeper into the intricate web of interactions involving SARS-CoV-2, we harnessed the power of STRING software (https://string-db.org/). This sophisticated computational tool facilitated an in-depth analysis of the virus's interactions with various other proteins, shedding light on the complex network of molecular associations that underlie its biology.

**Table 1.** Predictions by all supervised-based methods.

Variant ID	Mutations	SNAP2 score	SNAP2	Mutpred-2 score	Mutpred-2	SNPs&GO score	SNPs&GO	PhD SNP	SIFT score	SIFT	PROVEAN score	PROVEAN
QNV71204.1	N74K	-58	N	0.42	N	0.684	D	N	0.71	N	-1.309	N
QQG33753.1	R102S	-18	N	0.707	D	0.538	D	N	0.34	N	-0.160	N
QQG33753.1	A222V	-90	N	0.196	N	0.075	N	N	1.00	N	-0.096	N
QQL13968.1	D614G	-52	N	0.460	N	0.245	N	N	0.62	N	0.598	N
QQD86527.1	V622F	-11	N	0.415	N	0.282	N	N	0.10	N	-0.593	N
QQL14099.1	Q677H	-53	N	0.406	N	0.054	N	N	0.12	N	0.002	N
QQL14015.1	D1153G	22	D	0.471	N	0.204	N	N	0.02	D	-2.483	N
QQL14122.1	P1162S	-52	N	0.487	N	0.413	N	N	0.65	N	-2.722	D

### Prediction of missense SNPs by homology & supervised-based methods

The determination of the pathogenicity of missense single-nucleotide polymorphisms (SNPs) involves the application of various computational tools, each with its specific threshold values and criteria. Firstly, the SIFT algorithm employs a cut-off value of 0.05. When the SIFT score falls below this threshold, it signifies a deleterious effect of the SNP, indicating its potential to disrupt protein function. Conversely, when the score exceeds 0.05, the SNP is categorized as having a neutral impact on protein function.

Similarly, the PROVEAN algorithm employs a cut-off value of -2.00. SNPs with PROVEAN scores lower than this threshold are considered deleterious, indicating their potential to adversely affect protein function, while those with scores higher than -2.00 are classified as neutral SNPs, suggesting that they are less likely to cause significant functional perturbations.

To gain a more comprehensive understanding of the impact of these missense SNPs, we conducted a thorough evaluation using two sequence homology-based methods, SIFT and PROVEAN. This evaluation focused on eight specific missense variants, with the aim of assessing their potential pathogenicity or neutrality. We extended our analysis by incorporating four supervised-based prediction methods tailored for missense SNP prediction. These methods, namely SNAP2, Mut-Pred2, PhD-SNP and SNPs&Go, offer further insights into the potential consequences of these genetic variants. For SNAP2, a cut-off value of 0.1 is applied to distinguish between potentially pathogenic and neutral SNPs.

The MutPred2 score serves as a vital parameter in our evaluation. This score effectively gauges whether a given amino acid substitution is likely to be pathogenic or benign. An amino acid is deemed pathogenic if its MutPred2 score reaches or exceeds the threshold of 0.50, indicating a higher likelihood of contributing to a detrimental functional alteration. This comprehensive approach employs a suite of bioinformatic tools, each with its specific threshold and criteria, to rigorously assess the impact of missense SNPs on protein function and, by extension, their potential role in disease pathogenesis. These methodologies collectively provide a robust framework for understanding the genetic variations under scrutiny, aiding in the identification of variants with clinical significance. This endeavor contributes to the broader understanding of genetic predispositions and their implications for human health [5].

#### Protein kinases [^3-22^,^25^,^27^]

- Serine/threonine kinases: Enzymes that phosphorylate proteins on serine or threonine residues. Serine/threonine kinases are involved in many signaling pathways and are essential for the proper functioning of cells. Dysregulation of these kinases has been implicated in various diseases, including cancer and neurodegenerative disorders;
- Myotonin protein kinase (MT-PK): encoded by the *DMPK* gene, associated with myotonic muscular dystrophy. Functions in skeletal muscle maintenance, myocyte differentiation and more;
- Liver kinase B1 (LKB1): encoded by the *STK11* gene, linked to Peutz–Jeghers syndrome and various cancers. Involved in cell metabolism, apoptosis, DNA damage response and spermiogenesis;
- Checkpoint kinase 2 (CHEK2): encoded by the *CHEK2* gene, associated with Li–Fraumeni syndrome, prostate cancer and others. Regulates cell cycle, DNA repair and apoptosis;
- Ataxia-telangiectasia mutated kinase (ATM): encoded by the *ATM* gene and located on chromosome 11q22–23. Linked to ataxia-telangiectasia and various cancers. Functions as a DNA damage sensor, activates checkpoint signaling and has multiple roles in cellular processes;
- Mitogen-activated protein kinase activated protein kinase-1-B (MAPKAP-K1b): encoded by the *RPS6KA2* gene on chromosome 6q27. Associated with Coffin–Lowry syndrome, ovarian cancer and mental retardation. Regulates mitogenic processes, translation, cellular proliferation and acts as a tumor suppressor [20,22,24];
- Lim kinase-1 (LIMK1): encoded by the *LIMK1* gene on chromosome 7q11.23. Linked to Williams–Beuren syndrome and related conditions. Regulates cell motility, microtubule disassembly and phosphorylation of specific proteins;
- Adenosine Mono Phosphate-Activated Protein Kinase (AMPK): comprises multiple subunits encoded by various genes. Associated with a wide range of diseases. Regulates cellular energy, metabolism, lipid synthesis, insulin signaling and cellular polarity;
- Eukaryotic Initiation Factor 2A Kinase 3 (EIF2A-kinase 3): encoded by the *EIF2AK3* gene and implicated in Wolcott–Rallison syndrome, Menkes Syndrome and more. Activates the integrated stress response, controls mitochondrial function and adaptation to stress;
- Tyrosine-protein kinases: includes Janus kinase 3 (JAK3), Abelson tyrosine kinase (ABL1), and other receptor tyrosine kinases. Associated with immunodeficiency diseases, leukemia and various cellular processes such as cell growth, cytoskeleton dynamics and immune response;
- Receptor tyrosine kinases (RTKs): RET and MET receptors contribute to cell proliferation, differentiation and neuronal development. Mutations can lead to various diseases, including Hirschsprung's disease and cancer [21,24,25,27];
- Platelet-derived growth factor receptors (PDGFRA and PDGFRB) regulate cell proliferation, survival and chemotaxis. Mutations are associated with several neoplastic disorders;
- Anaplastic lymphoma kinase (ALK) influences nervous system development, energy regulation and cell growth. Linked to lymphomas and neuroblastoma;
- Insulin receptor kinase mediates insulin actions and phosphorylation of intracellular substrates. Implicated in diabetes and related syndromes;
- Protein tyrosine phosphatase, *MTM1*, plays roles in muscle maintenance, mitochondrial function and myopathy [20,24,25,27].

These protein kinases have diverse functions and are involved in various diseases and cellular processes, making them crucial targets for therapeutic intervention and further research. The brief information regarding the protein kinases are given in Supplementary Table 1.

### Interactions & mutations of the spike protein of SARS-CoV-2

In our quest to unravel the intricate web of interactions that define the behavior of SARS-CoV-2, we turned to the invaluable tool known as STRING viruses, which serves as a powerful platform for the comprehensive analysis of the virus's interactions with various other proteins [31]. This analytical approach allows us to gain deeper insights into the multifaceted molecular relationships that govern the virus's biology, shedding light on the complex network of associations that drive its pathogenic mechanisms [32,33].

One protein of notable interest within this intricate network is 7a, a non-structural protein that assumes a pivotal role in the virus's life cycle, particularly during the process of viral replication within cultured cells. This role is of paramount importance as it influences the virus's ability to propagate and establish infection within its host [34].

This study's approach, which combines genetic sequence analysis with protein–protein interaction exploration, provides valuable insights into the genetic diversity of SARS-CoV-2 spike protein sequences in the Pakistani population and sheds light on potential functional consequences of mutations, particularly in relation to viral replication and host immune responses. Further investigation and characterization of these mutations may contribute to our understanding of the virus's behavior and inform strategies for diagnostics and therapeutics [35]. The brief data are provided in Supplementary Files 2–4.

### Prediction of missense SNPs by homology & supervised-based methods

Homology-based and supervised-based methods are two different approaches used in bioinformatics and computational biology for predicting various molecular properties or biological outcomes, such as protein function or the impact of genetic variants. Here are the key differences between these two methods:

Homology-based methods (HBM) rely on the assumption that similar sequences or structures have similar functions. They compare the target sequence or structure to known sequences or structures in databases to infer its function or properties based on similarity.

Supervised-based methods (SBM) involve the training of machine learning models using labeled data (examples with known properties or outcomes). These models learn to make predictions based on patterns and features extracted from the data.

HBM require databases of known sequences or structures for comparison. They depend on the availability of closely related homologous sequences or structures.

SBM require labeled training data, which means datasets where the properties or outcomes of interest are already known. The quality and representativeness of training data are critical.

HBM are often used for tasks such as protein function prediction, protein structure prediction and identifying conserved motifs or domains in sequences. They work well when close homologs with known functions are available.

SBM are versatile and can be applied to various tasks, including protein–protein interaction prediction, disease classification based on genetic variants, drug target prediction and more. They can be used when labeled data is available, even if closely related homologs are not.

HBM tend to be limited to the information present in the database and may not generalize well to novel or highly divergent sequences or structures.

SBM have the potential to generalize beyond the training data, making them suitable for predicting properties or outcomes for sequences or instances not present in the training set.

HBM are generally simpler in concept and execution, involving sequence or structural alignments and similarity calculations.

SBM can be more complex and require expertise in machine learning, feature selection and model training. They may also require more computational resources.

HBM rely on sequence or structural similarity to make predictions, whereas supervised-based methods use machine learning models trained on labeled data to make predictions based on patterns. The choice between these methods depends on the specific bioinformatics task, the availability of data and the desired level of generalization [^21-30^].

The threshold criteria for variant pathogenicity prediction using the SIFT algorithm have been established at a stringent level of significance, denoted as 0.05. Below this critical threshold, variants receive a classification of ‘deleterious’, signifying a high likelihood of being functionally detrimental, while variants surpassing this threshold are deemed ‘neutral’ single-nucleotide polymorphisms (SNPs), indicating a reduced probability of functional impact. The PROVEAN algorithm employs a distinct threshold criterion, set at -2.00, to stratify variants based on their predicted impact. Variants with scores below this threshold are categorized as deleterious, whereas those surpassing it are classified as neutral SNPs.

In Table 1, the predictions for various mutations using supervised-based methods, including SNAP2, MutPred-2, SNPs&GO, PhD SNP, SIFT and PROVEAN. Mutations were identified by their Variant ID and corresponding amino acid changes. The variant N74K demonstrated the deleterious result in SNPs&GO Score as 0.684. Whereas the R102S showed the deleterious score via MutPred-2 Score as 0.707 and SNPs&GO Score as 0.538. The variants A222V, D614G, V622F, and Q677H did not show any deleterious traits. The pathogenic variant D1153G demonstrated the SNAP2 Score of 22, and SIFT Score of 0.02. Last, the variant P1162S was concluded as deleterious through the PROVEAN Score of -2.722.

In Table 1, we present the outcomes of predictions generated through a battery of supervised-based methods, which encompass SNAP2, MutPred-2, SNPs&GO, PhD SNP, SIFT and PROVEAN. These predictions have been associated with specific mutations, each identified by its Variant ID, and denoting the resultant amino acid changes. Among these mutations, the variant N74K has elicited notable attention due to its deleterious classification. This classification was primarily derived from the SNPs&GO Score, registering a value of 0.684. Conversely, for the mutation denoted as R102S, two distinct supervised-based methods, MutPred-2 and SNPs&GO, have indicated a deleterious potential. MutPred-2 assigned a score of 0.707, while SNPs&GO yielded a score of 0.538.

It is worth highlighting that the variants A222V, D614G, V622F, and Q677H did not exhibit any discernible deleterious characteristics across the spectrum of the employed methods, signifying their potential neutrality or limited impact. In stark contrast, the mutation D1153G emerged as a noteworthy pathogenic variant, as evidenced by its SNAP2 Score of 22 and a rather unfavorable SIFT Score of 0.02. These scores collectively suggest a potential detrimental effect associated with this genetic alteration.

The variant P1162S has been conclusively categorized as deleterious, substantiated by the PROVEAN Score, which registered a substantially negative value of -2.722. These findings collectively contribute valuable insights into the potential functional consequences of these mutations within the context of protein structure and function.

In the Table, ‘D’ signifies deleterious predictions, indicating that the mutation is likely to have a negative impact, while ‘N’ indicates neutral predictions, suggesting that the mutation may not significantly affect protein function. These predictions offer insights into the potential consequences of these mutations on protein structure and function.

The pathogenicity assessment of eight missense variants was conducted using two sequence homology-based methodologies, specifically SIFT and PROVEAN. Subsequently, an additional evaluation was undertaken utilizing four supervised machine learning-based prediction algorithms tailored for missense SNP classification, which encompass SNAP2, Mut-Pred2, PhD-SNP and SNPs&Go. It is worth noting that SNAP2 employs a distinct threshold, characterized as 0.1, to segregate variants into pathogenic and non-pathogenic categories. Moreover, the MutPred2 algorithm provides a quantitative score that signifies the likelihood of an amino acid substitution being pathogenic. A score of 0.50 is employed as the critical threshold, with values equal to or exceeding this threshold indicative of the amino acid substitution's pathogenic potential, as delineated in Table 1.

### Nucleocapsid protein of SARS-COV-2

In addition to the spike protein, the nucleocapsid protein plays a pivotal role in the structural composition of the coronavirus. This protein exhibits a well-defined amino acid sequence comprising 419 residues, characterized by the presence of five distinct domains within its structural framework. To elucidate the variations within the nucleocapsid protein sequence, we sourced data from the National Center for Biotechnology Information (NCBI) specifically for the Pakistani population. Subsequently, an exhaustive analysis was conducted to identify and scrutinize the mutations present within these sequences. By meticulously examining a cohort of 100 nucleocapsid protein sequences, we successfully pinpointed 19 instances of mutations occurring within this critical protein. These mutated sequences were subjected to a comprehensive battery of computational tools, which were applied in conjunction with both the wild-type and mutated nucleocapsid protein sequences. In the context of these investigations, five model ligands were utilized to probe the binding affinities associated with the wild-type and mutated variants of the nucleocapsid protein. The binding interactions between these ligands and the nucleocapsid protein sequences, whether wild-type or bearing mutations, were systematically assessed. Remarkably, all tested ligands exhibited discernible binding affinities with both the mutated and wild-type nucleocapsid protein sequences, as presented in Table 1. These findings underscore the intricate molecular interactions underpinning the functionality of the nucleocapsid protein and its potential relevance in the context of viral pathogenesis and therapeutics.

In Table 2, the heat map exhibits a gradient of coloration, with the dark red region representing genetic variants that exhibit robust binding affinity to the molecules Berberine and Disogen, characterized by binding energies surpassing the threshold of -8.1 kcal/mol. In contrast, the light pink region denotes variants that display comparatively weaker binding interactions, with binding energies measured at -7.7 and -7.9 kcal/mol. This diminished binding is observed in association with the compounds Curcumin and Emodin. The regions colored in light and dark blue on the heat map correspond to genetic variants with the least favorable binding energies, ranging from -7.1 to -7.7 kcal/mol. This indicates a substantially weaker interaction with Berberine, Disogen, Curcumin and Emodin when compared with the aforementioned dark red and light pink regions, thus signifying a lower binding propensity in these cases.

**Table 2.** Binding affinities (kcal/mol) of five model drugs against wild and mutated nucleocapsid protein of SARS-COV-2.

Mutation	Berberine	Disogen	Curcumin	Apigenin	Emodin
Normal	-8.19	-8.218	-7.674	-7.184	-7.529
R209I	-8.191	-8.22	-7.789	-7.531	-7.531
S194L	-8.191	-8.215	-7.927	-7.343	-7.876
S188P	-8.192	-8.22	-7.587	-7.538	-7.518
R195I	-8.19	-8.214	-7.584	-7.393	-7.539
S202N	-8.191	-8.215	-7.594	-7.537	-7.537
T205I	-8.191	-8.215	-7.703	-7.217	-7.524
S327L	-8.19	-8.216	-7.571	-7.152	-7.528
T379I	-8.192	-8.213	-7.594	-7.172	-7.537
S413I	-8.192	-8.213	-7.567	-7.328	-7.533
R14H	-8.191	-8.216	-7.572	-7.233	-7.538
P13L	-8.21	-8.132	-7.684	-7.342	-7.54
Q289H	-8.143	-8.243	-7.688	-7.986	-7.808
R203K	-8.176	-8.312	-7.599	-7.845	-7.519
G204R	-8.178	-8.218	-7.192	-7.134	-7.891
D348Y	-8.156	-8.21	-7.905	-7.685	-7.543
K374T	-8.176	-8.206	-7.13	-7.523	-7.578
D377Y	-8.139	-8.214	-7.562	-7.034	-7.599
D402Y	-8.172	-8.208	-7.192	-7.976	-7.521
D03Y	-8.141	-8.217	-7.987	-7.623	-7.532

### Prediction of missense SNPs by MUpro, PROVEAN & POLYPHEN-2

In our meticulous assessment of mutations and their potential effects on protein stability, we employ various scoring systems, each offering nuanced insights into the impact of genetic variations. First, a score of zero serves as a critical threshold, signaling that a mutation has indeed compromised the stability of the protein under examination. The magnitude of this score holds significance, as a smaller score signifies a more substantial decrease in predicted protein stability. In contrast, a higher score on this scale would suggest a comparatively milder impact on protein stability. This scale, often utilized in the context of MUpro, allows us to gauge the degree of destabilization caused by specific mutations, providing valuable information for understanding their functional consequences.

Moving to PROVEAN, a distinct set of criteria comes into play. Here, the cut-off value is set at -2.5, and this threshold demarcates the line between deleterious and normal mutations. Mutations yielding a PROVEAN score equal to or below -2.5 are categorized as deleterious, indicating their potential to disrupt protein function. Conversely, mutations with scores above this threshold are considered normal, suggesting a lesser likelihood of causing significant functional disturbances. This specific scoring system offers a binary classification that aids in the rapid identification of mutations with potential clinical significance. POLYPHEN-2, on the other hand, introduces a score range that spans from 0 to 1. Within this framework, a score nearing 1 signifies that the mutation is likely to be damaging, with the potential to perturb protein function. Conversely, a score approaching 0 suggests that the mutation is more likely to be benign, with a reduced likelihood of causing significant harm to protein stability or function. POLYPHEN-2's scoring system is particularly useful for discerning the nuanced impact of mutations, allowing for a finer-grained assessment of their potential consequences. The brief data are provided in Supplementary Files 5–7.

These distinct scoring systems collectively enrich our understanding of the effects of mutations on protein stability and function. They offer a diverse array of criteria, from binary classifications to nuanced gradations, providing researchers and clinicians with valuable tools for characterizing genetic variants and their implications for health and disease. The presented data in the Table 3 provides a detailed account of genetic mutations, each associated with its unique accession number. These mutations have been subjected to comprehensive computational analyses using three distinct prediction tools: MUpro, PROVEAN and POLYPHEN-2, which are renowned for their efficacy in assessing the potential functional impacts of genetic variations.

**Table 3.** Mutations in the nucleocapsid protein in Pakistan.

Accession no.	Mutation	MU^pro^	PROVEAN	POLYPHEN-2
QQL14284.1	R209I	-0.3569479	-2.455	0.998
QQL14165.1	S194L	-0.67079386	-4.272	0.994
QQH16682.1	G204R	-0.68136619	-1.656	1.000
QQH15938.1	R203K	-1.0587625	-1.604	0.969
QQH17450.1	S188P	-1.2061166	-2.918	0.994
QQH16058.1	R1951	-0.6286178	-3.993	0.101
QQ16646.1	S202N	-0.67746599	-0.404	0.994
QPB18048.1	T2051	-0.24030545	-1.562	0.000
QQH16970.1	Q289H	-0.39194072	-1.270	0.994
QQH17450.1	S327L	0.55558303	-3.022	0.000
QQL14308.1	T379I	-0.3859215	-0.648	0.000
QQH15866.1	D348Y	-0.99177863	-0.588	1.000
QQH16526.1	K374T	-1.1104731	-0.819	0.997
QQH17294.1	D377Y	-0.42988461	-1.779	1.000
QQH16634.1	S413I	-0.29679539	0.311	0.998
QQL14047.1	D402Y	-0.59573415	-1.449	1.000
QQH16058.1	R14H	-0.81985283	-0.609	0.998
QQH16034.1	P13L	-0.79830902	-1.230	1.000
QQH16778.1	D03Y	-0.63221612	-0.103	1.000

For the mutation denoted as R209I (accession no: QQL14284.1), the MUpro score registers at -0.3569479, indicating a potential alteration in protein stability. Concurrently, PROVEAN assigns a score of -2.455, signifying a likely deleterious effect, while POLYPHEN-2 offers a score of 0.998, suggesting a high probability of this mutation causing functional changes. In the case of the mutation S194L (accession no: QQL14165.1), MUpro yields a score of -0.67079386, hinting at potential structural modifications in the protein. PROVEAN assigns a substantially negative score of -4.272, strongly implicating its detrimental nature, while POLYPHEN-2 provides a score of 0.994, indicating a high likelihood of functional impact. Mutation G204R (accession no: QQH16682.1) exhibits a MUpro score of -0.68136619, implying potential alterations in protein stability. PROVEAN assigns a score of -1.656, suggesting a moderate likelihood of deleterious effects, while POLYPHEN-2 assigns a score of 1.000, indicating a strong probability of functional alteration. These analyses continue for each mutation in the Table 3, providing valuable insights into their potential implications on protein structure and function.

Using PubMed.gov, we were able to zero down on the primary proteins engaged in COVID-19 and the potential drug targets for treating the infection. Data on protein–protein interactions were retrieved from the STRING database (https://string-db.org/), and an interaction network was designed to include both the proteins of central importance in COVID-19 and its therapeutic targets. This information was gathered from a variety of sources, including databases, text mining, neighborhood, occurrence, gene fusion, experiments and co-expression and a threshold of 70% for high confidence interaction scores as previously reported [5]. The results obtained after a thorough literature review resulted in a total of 14,289 phospho-sites found on 2703 host human phospho-proteins, 8471 quantified host human proteins, eight SARS-CoV-2 proteins, and two viral phospho-proteins [8]. According to a study, host human lung adenocarcinoma A549 cells expressing ACE2 exhibited 16,399 identified phospho-sites, constituting 4643 significant alterations. Phospho-proteomics was studied in iAT2s infected with SARS-CoV-2. Studying iAT2s is considered more feasible instead of AT2s since AT2s are quite hard to keep active in culture [3,4,^6-8^]. Notably, the AT2 transcriptional program and the capacity for self-renewal are maintained in iAT2 cultures at ALI [8].

**Figure 1.**
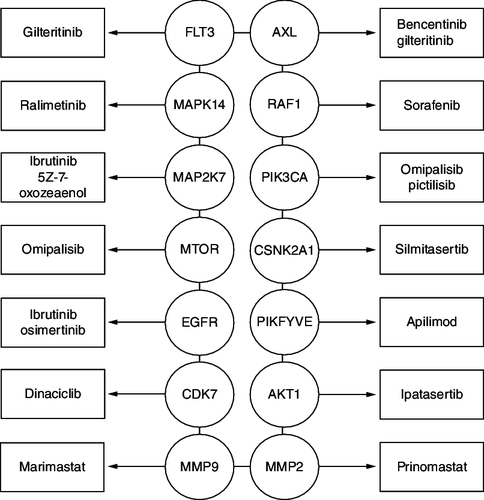
Therapeutic targets identified through interaction networks, artificial intelligence and machine learning.

Ralimetinib and ARRY-797 are undergoing phase III clinical trials for the treatment of cardiomyopathy, while Ralimetinib is undergoing phase II clinical trials for the treatment of ovarian cancer [22]. Bencentinib, another AXL inhibitor supports the antiviral activity seen for gilteritinib, an FDA-approved drug for treating acute myeloid leukemia. Ras/ERK, PI3K and p38 are among the intracellular signaling pathways that AXL is known to modulate; AXL inhibition in this case may help to downregulate p38 signaling (Figure 1). In a recent investigation [4], the PIKFYVE inhibitor apilimod was found to exhibit antiviral properties. They further develop this into a regulatory mechanism involving PIKFYVE phosphorylation in response to SARS-CoV-2 infection [4].

Around 4624 phosphosites in 3036 phosphoproteins were found in the chimpanzee Vero E6 cell line infected with SARS-CoV-2 [4]. In another study, Caco-2 cells from human colon cancer exhibited 15,093 phospho-sites in 7150 phospho-proteins [^8-10^]. Significant changes were also seen in 16,715 phosphopeptides after infection, with an increment of 2197 and decreases of 799, respectively. The protein levels in SARS-CoV-2-infected humans remain constant despite signaling events and differential phospho-protein modifying activities. This occurs even though more than 2000 phosphopeptides are generated during infection.

Hyper kinase activation and significantly increased phosphorylation are brought on by alterations in signaling activity. Proteins produced by viruses may be phosphorylated by kinases produced in the human host. To phosphorylate and generate substantial quantities of viral protein [3], SARS-CoV-2 exploits host cell enzymes. The gilteritinib and RAF, omipalisib, RO5126766, sorafenib, MEK or MAP2K2 and pictilisib are all possible treatments for COVID-19. These inhibitors, either alone or in combination, could be used to treat the ongoing COVID-19 pandemic [7].

The viral replication of SARS-CoV-2 has been considered to be significantly inhibited by the use of the antiviral drugs gilteritinib, ipatasertib, prinomastat and marimastat, with no appreciable effect on cell growth. The gilteritinib blocks FLT3 and AXL while ipatasertib blocks AKT. The antiviral effects of tirapazamine and gilteritinib against SARS-CoV and SARS-CoV-2 were reported [6]. Prinomastat has been shown to inhibit MMP2 whereas marimastat has been shown to inhibit MMP9 [3,21]. Interestingly, the MMP inhibitors greatly decreased SARS-CoV-2 viral activity but had little effect on SARS-CoV. The hallmarks of COVID-19 like TGF activation, neuroinflammation, alveolar injury and pleural effusions are related to MMP activities [4].

Our study regarding spike and nucleocapsid protein found deleterious mutations causing molecular anomalies in the human body. These incongruities could be treated through anti-sense technology. These antisense moieties are delivered to the body through organic (dendrimer, polymer, lipid)/inorganic (gold, silver, copper), lipid, synthetic/polymeric nanoparticles, aerosol system or intratracheal administration and cationic liposomes are used for the delivery of siRNAs [9].

We discovered drugs that inhibit PIKFYVE, p38 MAPK signaling, CK2 and CDKs exhibiting potent antiviral activity. After SARS-CoV-2 infection and pre-treatment with inhibitor compounds, viral load (as measured by an anti-NP antibody against SARS-CoV-2) and cell survival were determined post-48 h. Remdesivir was evaluated as a positive control for comparison and the possible effective antiviral activity was observed (half maximum inhibitory concentration [IC_50_] = 1.28 mM]) [3,19,23].

The CSNK2A1 and CSNK2A2 inhibitor silmitasertib was discovered to have antiviral action (IC_50_ = 2.34 mM). CK2 signaling appears to be a significant mechanism that SARS-CoV-2 has hijacked in light of the evidence for physical contact, co-localization with N protein and potential involvement in alterations to the extracellular matrix. Furthermore, silmitasertib is being investigated in human clinical trials as a prospective COVID-19 treatment option [4].

Of the 68 drugs and substances discovered, antiviral activity for a number of them that are FDA-approved in clinical trials, or preclinical development for various diseases, such as ARRY-797 (phase II/3, p38), SB203580 (preclinical, p38), silmitasertib (phase II, CK2), MAPK13-IN-1 (preclinical, p38), gilteritinib (AXL, FDA approved), ralimetin. To tackle COVID-19, silmitasertib, a tiny chemical undergoing clinical trials for several malignancies, is now being investigated for testing in human subjects. Although Gordon *et al.* [22] suggested that CK2 modulation of stress granules may be responsible for the efficiency of CK2 inhibition; CK2-mediated remodeling of the extracellular matrix may promote viral egress and spread [23]. Synthetic vehicles-ribonucleic acid delivery – after using very stable chemistries, (siRNAs) and (ADAR-oligonucleotides) can be changed or modified, messenger ribonucleic acid-based and deoxyribonucleic acid-based therapeutics need some medium for entrance in cells [3,9,23]. Different RNA systems of delivery are now introduced for this purpose. By using materials like (polymers and lipid nanoparticles) it is usually done. LNPs are with many nanoparticles. For liver siRNA delivery it is approved by FDA and for mRNA vaccine delivery too.

Studies showed that polymers deliver RNA in the cells. Polylactic co-glycol acid is FDA approved for small-drug molecule delivery. It is not recommended for the delivery of nucleic acid. (PLGA) is used along with chitosan for the delivery of siRNA in mice. FDA-approved lipid nanoparticles (LNPs) – these LNPS are having with variations of four components including (a cationic lipid), (cholesterol), (hyper lipid) and (PEG)-polyethylene glycol). Delivery is influenced by the structures of lipids. Many studies show the experiments of successful siRNA delivery in hepatocytes of mice [28].

## Discussion

In our analysis, we harnessed STRING to probe the interactions involving SARS-CoV-2 proteins. We conducted a sequence analysis of 60 spike protein sequences specifically sourced from Pakistan, obtained from the National Center for Biotechnology Information (NCBI) [31,32]. Through multiple sequence alignment, we pinpointed eight distinct mutation sites within these sequences. One notable finding was the identification of the non-structural protein 7a, which plays a crucial role in virus replication in cell cultures. Furthermore, we observed that LY75, also known as lymphocyte antigen 75, serves as an endocytic receptor involved in directing captured antigens from the extracellular space to the antigen-processing compartment. These insights into protein interactions and mutations are valuable contributions to the broader understanding of SARS-CoV-2 behavior and potential therapeutic strategies [^33-35^].

Our study underscores the significance of integrating bioinformatics resources like STRING with sequence analysis to gain insights into the molecular intricacies of viral proteins and their interactions with host proteins. It highlights the importance of studying regional variations in viral sequences, as evidenced by the specific focus on sequences from Pakistan. These findings can inform future research efforts aimed at developing targeted interventions against SARS-CoV-2 [^36-40^].

In our study, we employed several computational tools and methods to assess the potential impact of missense single-nucleotide polymorphisms (SNPs) on protein function. Two commonly used sequence homology-based methods, SIFT and PROVEAN, were employed with specific cut-off values (0.05 for SIFT and -2.00 for PROVEAN). These cut-off values help classify the SNPs as deleterious (below 0.05 for SIFT) or neutral (above 0.05 for SIFT). To ensure a comprehensive evaluation, we extended our analysis to include four supervised-based prediction methods specifically designed for missense SNP prediction: SNAP2, Mut-Pred2, PhD-SNP and SNPs&Go. For SNAP2, we used a cut-off value of 0.1 [37,41].

Our approach is in line with contemporary research strategies aimed at comprehensively evaluating missense SNPs to ascertain their potential functional implications. By incorporating multiple prediction tools with varying algorithms and cut-off values, we sought to provide a robust assessment of these genetic variations [42].

This approach enhances the reliability of predictions and reduces the risk of false positives or false negatives. Comparing our results with those of other studies utilizing similar tools and thresholds will contribute to a more comprehensive understanding of the potential impact of missense SNPs on protein function and their relevance to disease susceptibility [43,44].

In our study, we focused on the nucleocapsid protein of SARS-CoV-2, recognizing its significance alongside the well-studied spike protein. The nucleocapsid protein is crucial as it plays a role in covering the virus's surface and is integral to its structure [45]. This protein comprises an amino acid sequence of 419 residues and possesses five distinct domains within its structure. To gain insights into the genetic variations of the nucleocapsid protein in the Pakistani population, we extracted its sequences from the National Center for Biotechnology Information (NCBI) database and subsequently analyzed these sequences for mutations. Among the 100 nucleocapsid protein sequences examined, 19 were found to exhibit mutations [46,47].

To understand the potential impact of these mutations, we employed various computational tools and applied them to both wild-type and mutated nucleocapsid protein sequences. In particular, we assessed the binding affinities of five model ligands to these protein sequences. Our analysis revealed that all of the ligands displayed affinities for both the mutated and wild-type nucleocapsid protein sequences, as outlined in Table 2. The brief data regarding their energy attributes are provided in Supplementary Files 8–12.

Comparing our findings with other relevant studies in the field, we acknowledge that the nucleocapsid protein's role in SARS-CoV-2 has gained increasing attention due to its involvement in viral structure and function. Several studies have explored the genetic diversity of this protein, as genetic variations can potentially impact viral behavior and host interactions [48].

Our study contributes to this body of research by specifically investigating the nucleocapsid protein in the context of the Pakistani population, shedding light on the prevalence of mutations and their potential effects on protein-ligand interactions. These findings add to the growing understanding of the genetic diversity of SARS-CoV-2 and may have implications for antiviral drug development and therapeutic strategies. While our study provides valuable insights, it is essential to recognize that the nucleocapsid protein's multifaceted role in viral biology continues to be an active area of research. Future studies can build upon our findings and further investigate the functional consequences of these mutations and their implications for viral pathogenesis and immune response. Collaborative efforts across multiple studies will contribute to a comprehensive understanding of SARS-CoV-2 and enhance our ability to combat the virus effectively [^49-52^].

In our study, we investigated the interactions of SARS-CoV-2 proteins with other proteins using the STRING viruses' database. Specifically, we identified two proteins of interest, namely 7a and LY75 (Lymphocytic antigen 75), and explored their potential roles in the viral life cycle and host immune response. The non-structural protein 7a, as identified in our analysis, has been previously recognized as a key player in the replication of viruses in cell cultures. This finding aligns with existing research that has highlighted the significance of this protein in the context of viral replication. Understanding the role of 7a in the SARS-CoV-2 life cycle can aid in the development of targeted antiviral strategies.

Our study shed light on the role of LY75, also known as Lymphocyte antigen 75, in the context of SARS-CoV-2 infection. LY75 functions as an endocytic receptor, directing captured antigens from the extracellular space to the antigen-processing compartment. This mechanism can be crucial in the context of the host immune response to the virus. While our study provides insights into the potential involvement of LY75 in SARS-CoV-2 infection, further research is needed to fully elucidate its role in the immune response to the virus [^40-43^].

Comparing our findings with existing literature, we find support for the role of 7a in viral replication, which is consistent with previous research on this protein in the context of coronaviruses. However, the specific involvement of LY75 in SARS-CoV-2 infection and its implications for the immune response require further investigation and validation through experimental studies [44,45].

Our study underscores the importance of utilizing bioinformatics resources like STRING viruses to uncover potential protein interactions in viral infections. These findings contribute to the growing body of knowledge regarding the molecular mechanisms of SARS-CoV-2 and may inform future research efforts aimed at understanding the virus-host interactions and developing targeted interventions. Collaborative efforts with other studies focusing on protein interactions in SARS-CoV-2 can provide a more comprehensive understanding of the virus's biology and pathogenesis [46,47].

In our study, we employed various computational tools to assess the potential impact of mutations on protein stability and function. These tools, including MUpro, PROVEAN, and POLYPHEN-2, play a crucial role in predicting the consequences of genetic variations, offering valuable insights into the deleterious or benign nature of mutations. MUpro utilizes a scoring system where a zero value signifies that the mutation has resulted in a decrease in protein stability. The smaller the score, the greater the predicted decrease in protein stability, indicating the potential detrimental effect of the mutation [48,49].

Comparing our results with those of other studies, it's evident that these tools are essential for prioritizing and understanding the impact of genetic variations. Researchers frequently rely on similar scoring systems to assess the pathogenicity of mutations and their relevance to disease. It's important to note that the choice of cut-off values and scoring systems can vary between studies and depend on the specific research objectives. Therefore, the interpretation of mutation data should consider both the computational tools used and the chosen thresholds [^50-54^]. Experimental validation is often required to confirm the functional effects of mutations predicted by these tools. Our study's utilization of these computational tools adds to the body of research aimed at characterizing genetic variations and their implications for protein stability and function. Collaborative efforts and comparisons with other studies that employ similar tools can provide a more comprehensive understanding of the functional consequences of mutations and their role in disease development [55].

Future studies can further validate our findings and expand upon them by considering additional prediction tools and larger datasets for a more in-depth analysis of missense SNPs.

## Limitations & future perspective

The training data used for these tools represents a diverse range of proteins and their functional categories. Biased training data can lead to inaccurate predictions on proteins that differ significantly from the training set. We did target only those proteins that were previously reported to have strong foundations. However, we assessed the performance of these tools on a diverse set of proteins or SNPs. If they consistently perform poorly on certain types of proteins or SNPs, it may indicate bias in the training data. External validation and transparency in data sources can help mitigate bias and improve the reliability of predictions. That's why we reported all the data of all the interactions in the Supplementary Section. There are numerous validation studies and benchmarks that have evaluated the tools' performance on diverse datasets. That's why we selected these models for our research to avoid false positive results. However, researchers should examine the source of training data and the criteria used to label SNPs as deleterious or neutral. Transparency in data sources and labeling criteria can help identify potential sources of bias.

While the methods described in the manuscript provide valuable tools for predicting the impact of SNPs on protein function, the potential for bias in predictions exists if the training data is not representative of the diversity of proteins and SNPs. Researchers should be cautious when interpreting results and consider the limitations associated with the training data used for these tools.

## Conclusion

Ensembl-based computational tools were employed to scrutinize a dataset encompassing 60 variants, and their collective predictions identified eight missense variants, namely N74K, R102S, A222V, D614G, V622F, Q677H, D1153G and P1162S. Subsequently, a multifaceted array of variant pathogenicity prediction methods was systematically applied, consisting of sequence homology-based and supervised-based approaches, as well as structure-based methodologies. Among the sequence homology-based techniques, D1153G demonstrated as deleterious for SNAP2 and SIFT; while PROVEAN confirmed P1162S as deleterious. The remaining variants received a neutral classification. Supervised-based methods, including SNAP2, MutPred2, PhD-SNP and SNPs&Go, further refined the variant assessment. SNAP2 identified D1153G as affected, with the remaining seven variants considered neutral. MutPred2 singled out R102S as damaging, while the other seven variants were predicted to be neutral. PhD-SNP, on the other hand, categorized all variants as neutral. SNPs&Go identified two variants, N74K and R102S, as disease-associated, while the remaining six were classified as neutral. Moving to structure-based methods, which encompassed MetaSNP, Predict-SNP, and PolyPhen-2, Predict-SNP identified D1153G as deleterious and the other seven as neutral. MetaSNP, however, classified all variants as neutral, while PolyPhen-2 pinpointed four variants, namely R102S, Q677H, D1153G and P1162S, as potentially damaging. In the context of viral pathogenesis, it is noteworthy that host human kinases play a crucial role in phosphorylating viral proteins, including those of SARS-CoV-2. This phosphorylation process leads to hyperkinase activation and significantly increased phosphorylation levels, contributing to viral protein production. Regarding potential therapeutics for COVID-19, several candidates have been identified, including Gilteritinib (a specific MMP9 inhibitor), Pictilisib (targeting PI3K), Sorafenib (inhibiting RAF and growth factor receptors), RO5126766 (involved in inhibiting RAF and both MEK or MAP2K2), and Omipalisib (an inhibitor of mTOR and PI3K). These inhibitors hold promise as viable and recommended treatment options for the ongoing COVID-19 pandemic, either as standalone therapies or in combination regimens, owing to their potential to disrupt critical signaling pathways and viral protein production.

## Supplementary Material

Supplementary Tables S1-S12
