# Supplementary material for: SARS-CoV-2-induced phosphorylation and its pharmacotherapy backed by artificial intelligence and machine learning
Source: Future Sci OA. 2024 May 15;10(1):FSO917. doi: 10.2144/fsoa-2023-0112 (PMC11140666; doi:10.2144/fsoa-2023-0112)
Supplement: Supplementary Tables S1-S12 [file IFSO_A_2341478_SM0001.docx]

**Supplementary File**

Table S1. The anomalies in the regulation of protein kinases and phosphatases can manifest serious health implications [1-25].

| **PROTEIN KINASES** |  | **Protein kinases/Phosphatase** | **Other names/Aliases** | | | **Encoded by the gene/ Protein attributes** | **Cytogenetic location** | **Diseases caused** | **Function/Phenomena involved** |
| --- | --- | --- | --- | --- | --- | --- | --- | --- | --- |
|  |  | **SERINE/THREONINE KINASES** | | | | | | | |
|  |  | Myotonin-protein kinase(MT-PK) | Dystrophia myotonica Protein Kinase (DMPK)/ myotonic dystrophy Protein kinase (MDPK)  **Domain:**  **Family:** AGC Ser/Thr protein kinase  **Subfamily**: DMPK | **Encoded by:** DMPK gene  **Size of the Protein:** 629 a.a  **Molecular mass:**  69385 Da | | | chromosome 19q13.3. | Myotonic muscular dystrophy | - Role in skeletal muscle maintenance, - Myocyte differentiation, - inhibit myosin phosphatase activity, - cardiac contractility modulation, - regulate the expression of muscle-related genes and the intactness of the nuclear envelope |
|  |  | Liver kinase B1(LKB1) | Human Tumor Suppressor LKB1; Serine/Threonine Protein Kinase 11 - STK11  **Family:** CAMK Ser/Thr protein Kinase  **Sub Family:** LKB1 | **Encoded by: STK**11 gene  **Size of the Protein**: 436 a.a.  **Molecular mass:**  48636 Da | | | chromosome 19p13. | Peutz–Jeghers syndrome, Testicular Carcinoma, Cutaneous Malignant, Melanoma | - Plays a role in cell metabolism, and cell polarity through actin cytoskeleton remodeling, apoptosis, and DNA damage response mainly through the interaction with p53/TP53 and recruitment to CDKN1A/WAF1 promoter for the transcription mediation. - Phosphorylates both AMPK and Non-AMPK proteins - Actively involved in spermiogenesis. - During carcinogenesis, the gene is known to be inactivated through mutation. Several mutations have been found and most cluster to the kinase domain or outside resulting in the loss or reduction of STK11 kinase activity |
|  |  | Check point kinase2 (CHECK2) | CHEK2,CHK2,CDS1; Serine/Threonine-Protein Kinase  **Family:** CAMK Ser/Thr Protein Kinase  **Subfamily:** CHK2 | **Encoded by:** CHEK2 (CDS1, CHK2, RAD53)  **Size of the Protein**: 543 a.a  **Molecular mass:**  60915 Da | | | chromosome 22q12.1 | Li–Fraumeni syndrome  Prostate cancer, Breast Cancer, Osteogenic Sarcoma, Colorectal cancer | - Involved in cell cycle arrest, mediated *via* checkpoint - Contributes to the DNA repair ,activation and apoptosis - During the unperturbed cell cycle. may negatively regulate the cell cycle progression - Regulates DNA repair through phosphorylation, - Tumor suppressor may cause chromosomal instability in cancer cells if absent. |
| **PROTEIN KINASES** |  | Ataxia- telangiectasia mutated kinase (ATM) | ATM Serine/Threonine Kinase, Serine-Protein Kinase ATM, Ataxia Telangiectasia Mutated **Family:** PI3/PI4-kinase  **Subfamily**: ATM | **Encoded by:** ATM gene  **Size:** 3056 a.a  **Molecular mass:**  350687 Da | | | chromosome 11q22–23 | Ataxia-telangiectasia, Pro-lymphocytic Leukemia of T cells, Mantle Cell Lymphoma | - Functions as a DNA damage sensor, and is known to activate checkpoint signaling and induce apoptosis and genotoxic stresses. - Involved in pre-B cell allelic exclusion, - ABL1 and SAPK activator. - Tumor suppressing role. - Multi-functional status as vesicle and/or protein transporter, T cell development enhancer as well as involved in neurological functions. - Due to the germ line mutation of the A-T, the mutated (ATM) gene causes Ataxia-telangiectasia.   . |
|  |  | Mitogen-Activated protein kinase activated Protein kinase -1-B (MiogeMAPKAP-K1b) | (MiogeMAPKAP-K1b)  1C, P90-RSK 2  **Famil**y: CMGC Ser/Thr protein kinase.  **Subfamily**: MAP kinase | **Encoded by:** RPS6KA2 Gene (Ribosomal Protein S6 Kinase A2)  **Size of the Protein:** 733 a.a  **Molecular mass:**  83239 Da | | | Chromosome 6q27 | Coffin-Lowry syndrome, Ovarian Cancer, Mental Retardation with Osteocartilaginous Abnormalities | - It functions as a mitogenic mediator and stress-induced activator of transcription factors - It regulates the process of translation and induces cellular proliferation, differentiation, and survival - Acts as a tumor suppressor |
|  |  | Lim kinase-1 – Enzyme protein encoded by LIMK1 | LIM Domain Kinase 1, LIMK1  **Family**: TKL Ser/Thr protein- kinase | **Encoded by:** LIMK1 Gene  **Size of the Protein:**647 a.a  **Molecular mass:**  72585 Da | | | Chromosome 7q11.23 | Williams-Beuren Syndrome, Supra-valvular Aortic Stenosis, Martin-Bell Syndrome | - Regulates several biological events *i.e.,* cell motility, cell cycle processes, and actin-dependent differentiation mechanisms. - Promotes disassembly of the microtubule. - Required for cofiilin’s (CFL1) atypical chemokine receptor ACKR2-induced phosphorylation. |
| **PROTEIN KINASES** |  | Adenosine Mono phosphate –activated protein kinase (AMPK) | 5' AMP-activated PK or AMPK, 5'  **PRKAA1 & PRKAA2**  **Family**: CAMK Ser/Thr protein kinase  **Subfamily:** SNF1  **PRKAG1, PRKAG2 & PRKAG3**  **Family**:  5'-AMP-activated PK gamma subunit | AMPK gene  **PRKAA1** Gene encodes the α1 protein,  **PRKAA2** Gene:  encodes the α2 protein  **PRKAB1** Gene: encodes β1 protein  **PRKAB2** Gene: encodes β2 protein  **PRKAG1** Gene: encodes the γ1 protein  **PRKAG2** Gene: encodes the γ2 protein, and  **PRKAG3** Gene: encodes the γ3 protein  **PRKAA1** Gene  **Size**:559 a.a  **Molecular mass:**  64009 Da  **PRKAA2** Gene  **Size:**552 a.a  **Molecular mass:**  62320 Da  **PRKAG1** Gene  **Size:**331 a.a  **Molecular mass:**  37579 Da  **PRKAG2** Gene  **Size:**569 a.a  **Molecular mass:** 63066 Da  **PRKAG3** Gene:  **Size:**489 a.a  **Molecular mass:**  **54258 Da** | | | **PRKAA1 Gene** 5p13.1,  **PRKAA2 Gene**  1p32.2  **PRKAG1 Gene**  12q13.12  **PRKAG2 Gene**  7q36.1  **PRKAG3 Gene**  2q35 | Wolff–Parkinson–White syndrome,  Myotonia, Cardia Cancer, Lung Cancer, Peutz-JeIhers Syndrome,  Tuberous Sclerosis, Thymus Lymphoma, Malignant Essential Hypertension, Cardiomyopathy, Familial, Malignant Hyperthermia | **AMPK:**   - Regulates cellular energy, metabolism (activation and inhibition of energy-producing pathways and energy-consuming processes respectively) - Participates in lipid synthesis - Functions in insulin-signaling and glycolysis, cell growth, and proliferation. - An important role in pro-autophagy and non-autophagy complexes’ differential regulation - In response to glucose starvation, it inhibits the non-autophagy complex - Regulator of cellular polarity through the remodeling of the actin cytoskeleton |
| **PROTEIN KINASES** |  | Eukaryotic initiation factor 2A Kinase 3 (EIF2A-kinase 3) | Eukaryotic Translation Initiation Factor 2 Alpha Kinase 3  **Family**: Ser/Thr protein kinase  **Subfamily:**  GCN2 | EIF2AK3 Gene (PEK, PERK),  **Size:**1116 a.a  **Molecular mass:**  125216 Da | | | chromosome 2p11.2 | Wolcott–Rallison syndrome,  Menkes Syndrome,  Multiple Epiphyseal Dysplasia | - Activator of the integrated stress response (ISR) - Required for adaptation to various stress, control of mitochondrial morphology and function |
|  |  | **TYROSINE-PROTEIN KINASES** | | | | | | | |
|  | **Non-Receptor Tyrosine Kinase (NPTK)** | JanusKinase3 (Nonreceptor Tyrosine kinase; JAK family) | JAK3-HUMAN; JAKL; Jack3 kinase (a protein tyrosine kinase leukocyte); JAKL; L-JAK; Leukocyte Janus kinase; LJAK  **Family**: Tyr protein kinase  **Subfamily:** JAK | | JAK3 Janus kinase 3  **Size of the Protein:**1124 a.a  **Molecular mass:**  125099 Da) | | Chromosome19p13.11 | X linked Severe Combined Immunodeficiency, Autosomal Recessive, T Cell-Negative, B Cell-Positive, Nk Cell-Negative, and Nk-Cell Enteropathy; Myeloproliferative disorders/ Neoplasm; Acute Megakaryocytic Leukemia | - Responsible for the major events of cell growth, development, or/and differentiation - It is mainly associated with the expression in the cells of the immune system and triggers a signal *via* tyrosine phosphorylation by interleukin receptors, as a response. - Mutations can lead to autosomal SCID (severe combined immunodeficiency disease). |
|  |  | Abelson tyrosine kinase (ABL1 non receptor TPK; ABL family) | Abl Kinase, C-Abl, ABL Proto-Oncogene 1, Non-Receptor Tyrosine Kinase  **Family**: Tyr protein kinase  **Subfamily**: ABL | | ABL1 gene  **Size of the Protein**:1130 a.a  **Molecular mass:**  122873 Da | | Chromosome 9q34.12 | Chronic myelogenous- leukemia, Chronic Myeloid Leukemia, Leukemia, Chronic Myeloid, Philadelphia Chromosome Positive, Somatic  Cml - Chronic Myelogenous Leukemia. T-Cell Acute Lymphoblastic Leukemia, B-Lymphoblastic Leukemia/Lymphoma With Recurrent Genetic Abnormality | - Regulates cell growth and survival. - Conforms the actin remodeling   *via* tyrosine phosphorylation of the proteins engaged in the dynamics of the cytoskeleton   - Triggers the late-stage of autophagy, - Targets mitochondria in reaction to oxidative stress - During an infection, regulate the multiple pathological signaling cascades |
|  | **Receptor Tyrosine Kinases (PTK)** | Zeta –associated protein 70, (Non receptor TPK; SYK family) | ZAP-70, STD,SRK, Zeta chain (TCR)-associated70kDa,TZK, ZAP70_Human,Zeta chain of T-cell receptor associated protein kinase 70, tyrosine phosphoprotein, Zeta  **Family:** Tyr protein kinase  **Sub Family**: SYK/ZAP-70 | | ZAP 70 gene  **Size of the Protein:** 619 a.a  **Molecular mass:**  69872 Da | | Chromosome 2q11.2 | Autosomal recessive SCID (suffer from severe combined immunodeficiency with dysfunctional T-cells in the peripheral blood) | - Regulates adaptive immune response - Controls the mature T-cells   motility and the processes of adhesion and cytokine expression   - Predominantly contributes to the development of thymocytes and primary B-lymphocytes. - Controls the activation and switching on and switch off of T-cells. - ZAP70-dependent signaling pathway contributes through B cell receptor in the primary B-cells formation and activation. - Mutations introduce selective T-cell aberrations, and a severe combined immune-deficiency issue attributed to the CD8-positive T-cell selective absence. |
| **PROTEIN KINASES**  **PROTEIN KINASES**  **PROTEIN KINASES** |  | Receptor Tyrosine kinase (RTKs); RET receptor family; RTK class XIV) | RET, RTK, (Ret Proto-Oncogene, Proto-Oncogene Tyrosine-Protein Kinase Receptor **Ret**  **Family**: Tyr protein kinase | | RET gene  **Size of the Protein:**1114 a.a  **Molecular mass:**  124319 Da | | Chromosome  10q11.21 | Hirschsprungı ́s disease, Thyroid Carcinoma, Familial, Multiple Endocrine Neoplasia, Type 2a | - Causes Cell proliferation, cell migration, and cell differentiation as well as neuronal navigation, - Regulates both cell mortality/survival balance and position-referenced information. - It contributes to neural crest development. - Promotes the differentiation of rapidly adapting (RA) mechanoreceptors. |
|  | **Receptor Tyrosine Kinases** | Mesenchymal-epithelial transition(Met) Receptor tyrosine kinase factor ( RTK) ( RTK class VIII) | Hepatocyte growth factor receptor (HGFR); MET Proto-Oncogene, Receptor Tyrosine Kinase  **Family**: Tyr protein kinase | | MET gene  **Size of the Protein:** 1390 a.a  **Molecular mass:**  155541 Da | | Chromosome 7q31.2 | Papillary renal cancer, Hepatocellular Carcinoma, Osteo-Fibrous Dysplasia | - Stewards the physiological process e.g., proliferation, scattering, morphogenesis, and survival - Plays a role during the process of gastrulation - Involved in the development and migration of neuronal and muscle precursors. - It directs the process of angiogenesis and kidney formation |
|  |  | Translocation-ETS-Leukemia virus (TEL)- Platelet derived growth factor (PDGF) receptor kinase ,  Receptor family, RTK class III) | TEL transcription factor which in humans is now encoded as ETV-6, Platelet-derived growth factor receptor alpha, Platelet Derived Growth Factor Receptor Beta,  **PDGFRA and PDGFRB**  **Family:** Tyr protein kinase  **Sub-family:** CSF-1/PDGF *receptor* | | **PDGFRA gene**:  **Size of the Protein**:1089 a.a  **Molecular mass:**  122670 Da  **PDGFRB gene:**  **Size of the Protein:**1106 a.a  **Molecular mass:**  123968 Da | | **PDGFRA**: 4q12  **PDGFRB**:  5q32 | Chronic myeloid-monocytic-leukemia, Chronic Eosinophilic Leukemia, Myeloid, and Lymphoid Neoplasms, Myeloproliferative Disorder, Gist-Plus Syndrome, Kosaki Overgrowth Syndrome,  Myofibromatosis, Infantile | **PDGFRA**   - Embryonic development regulator - Hampers the cell proliferation, survival as well as chemotaxis - Stimulates the migration and chemotaxis of the cells during wound healing, - Helps in the secretion of agonists from platelet granules, platelet activation and also promotes thrombin-induced platelet clustering,   **PDGFBRB**:   - Renders efficient role in the development of blood vessels. - Controls the migration of vascular smooth muscle cells and the formation of neo-intima at vascular injury sites. |
|  |  | Anaplastic Lymphoma kinase (ALK) | ALK Receptor Tyrosine Kinase, Cluster of differentiation 246( CD 246)  **Family**: Tyr protein kinase  **Sub Family**: Insulin receptor | | ALK gene:  **Size of the Protein***:* 1620 a.a  **Molecular mass:**  176442 Da | | 2p23.2-p23.1, | Alk-Positive Anaplastic Large Cell Lymphoma; Non-Hodgkins lymphoma, Neuroblastoma 3 | - An important role in the genesis and differentiation of the nervous system - Its action contributes to the resistance to weight gain   in hypothalamic neurons   - Activation of NF-kappa-B, essential for the mediation of autocrine growth - It serves to control energy expenditure through negative regulation of lipolysis of white adipose tissue. - It is an important protein that helps control growth of cells |
|  |  | Insulin receptor kinase | Insulin Receptor  **Family:** Tyr protein kinase. **Subfamily:** Insulin receptor | | INSR ( Gene encoding)  **Size of the Protein**:1382 a.a  **Molecular mass:**  156333 Da | | 19p13.2 | diabetes, Donohue Syndrome (Leprechaunism) Pineal Hyperplasia, Familial Hyper-insulinemic Hypoglycemia | - It has an essential role in mediating the pleiotropic actions of insulin. - Governs the phosphorylation of several intracellular substrates, the phosphorylated proteins in turn then serve as docking proteins for the signaling proteins that harbor Src-homology-2 domains (SH2 domain). This domain precisely helps to identify several different phosphotyrosine residues |
|  | **Protein Tyrosine Phosphatase** | MTM1Tyrophosphatase ,protein-tyrosine phosphatase (PTPases) | Myotubularin 1  **Family:** protein-tyrosine phosphatase  **Subfamily:** Non-receptor class myotubularin | | MTM1 gene  **Size of the Protein:** 603 a.a  **Molecular mass:**  69932 Da | | Xq28 | X-Linked Centro-nuclear Myopathy, X-Linked myotubular myopathy | - It is found engaged in vacuolar formation and morphology. - Regulates the desmin intermediate architecture and filament assembly - An essential role in mitochondrial morphology and positioning. - They play role in skeletal muscle maintenance but are not essential for myogenesis |

Table S2. Predictions by SIFT and PROVEAN score. Where ‘D’ stand for deleterious and ‘N’ for Neutral.

| **Variant ID** | **Mutations** | **SIFT SCORE** | **SIFT** | **PROVEAN SCORE** | **Outcome** |
| --- | --- | --- | --- | --- | --- |
| **QNV71204.1** | N74K | 0.71 | N | -1.309 | N |
| **QQG33753.1** | R102S | 0.34 | N | -0.160 | N |
| **QQG33753.1** | A222V | 1.00 | N | -0.096 | N |
| **QQL13968.1** | D614G | 0.62 | N | 0.598 | N |
| **QQD86527.1** | V622F | 0.10 | N | -0.593 | N |
| **QQL14099.1** | Q677H | 0.12 | N | 0.002 | N |
| **QQL14015.1** | D1153G | 0.02 | D | -2.483 | N |
| **QQL14122.1** | P1162S | 0.65 | N | -2.722 | D |

**Prediction of missense SNPs by Supervised based methods**

Four supervised-based computational methods were employed to assess and predict the potential impact of missense Single Nucleotide Polymorphisms (SNPs) in this study. These methods, namely SNAP2, MutPred2, PhD-SNP, and SNPs&Go, were selected to provide a comprehensive evaluation of the functional consequences of these genetic variations. Specifically, for SNAP2, a threshold of 0.1 was utilized as the cut-off value to distinguish between potentially pathogenic and benign missense SNPs. This threshold value was chosen to classify SNPs based on their predicted impact on protein function.

MutPred2, on the other hand, employs a scoring system to evaluate whether a specific amino acid substitution is likely to be pathogenic or not. In this context, a MutPred2 score of 0.50 serves as the critical threshold. If the score surpasses this threshold, it indicates a higher likelihood of the amino acid alteration being pathogenic, implying potential functional disruptions in the corresponding protein. The integration of these methods enables a robust assessment of missense SNPs, offering valuable insights into their potential deleterious effects on protein function and aiding in the identification of variants with clinical significance or implications in disease susceptibility.

Table S3. Predictions by all supervised based methods. D stand for deleterious and N for Neutral.

| **Variant ID** | **Mutations** | **SNAP2 score** | **SNAP2** | **Mutpred2 score** | **Mutpred-2** | **SNPs&GO**  **score** | **SNPs&GO** | **PhD SNP** |
| --- | --- | --- | --- | --- | --- | --- | --- | --- |
| **QNV71204.1** | N74K | -58 | N | 0.42 | N | 0.684 | D | N |
| **QQG33753.1** | R102S | -18 | N | 0.707 | D | 0.538 | D | N |
| **QQG33753.1** | A222V | -90 | N | 0.196 | N | 0.075 | N | N |
| **QQL13968.1** | D614G | -52 | N | 0.460 | N | 0.245 | N | N |
| **QQD86527.1** | V622F | -11 | N | 0.415 | N | 0.282 | N | N |
| **QQL14099.1** | Q677H | -53 | N | 0.406 | N | 0.054 | N | N |
| **QQL14015.1** | D1153G | 22 | D | 0.471 | N | 0.204 | N | N |
| **QQL14122.1** | P1162S | -52 | N | 0.487 | N | 0.413 | N | N |

Neutral.

**Consensus based methods**

Cut-off value for Meta-SNP is 0.5. Cut-off value of Poly-Phen is 0.5.

Table S4. Predictions of SNPs by consensus and structural based methods.

| **Variant ID** | **Mutations** | **Meta-SNP**  **score** | **Meta-SNP prediction** | **Predict-SNP-%** | **Predict-SNP prediction** | **Poly-Phen**  **score** | **Poly-Phen prediction** |
| --- | --- | --- | --- | --- | --- | --- | --- |
| **QNV71204.1** | N74K | 0.479 | Neutral | 65% | Neutral | 0.000 | Benign |
| **QQG33753.1** | R102S | 0.493 | Neutral | 65% | Neutral | 0.996 | Probably damaging |
| **QQG33753.1** | A222V | 0.041 | Neutral | 83% | Neutral | 0.000 | Benign |
| **QQL13968.1** | D614G | 0.227 | Neutral | 83% | Neutral | 0.004 | Benign |
| **QQD86527.1** | V622F | 0.244 | Neutral | 63% | Neutral | 0.116 | Benign |
| **QQL14099.1** | Q677H | 0.085 | Neutral | 63% | Neutral | 0.480 | Possibly damaging |
| **QQL14015.1** | D1153G | 0.266 | Neutral | 51% | Deleterious | 0.978 | Possibly damaging |
| **QQL14122.1** | P1162S | 0.235 | Neutral | 74% | Neutral | 0.936 | Possibly damaging |

**Nucleocapsid protein of SARS-COV-2.**

**Prediction of missense SNPS by MU pro:**

A score with zero value indicate that mutation has decreased the stability of the protein. The smaller the score, the more predicted decrease in protein stability and vice versa.

Table S5. The mutations in different variants of SARS-COV-2. Where ‘D’ stand for deleterious and ‘N’ for Neutral.

| **Accession no** | **Mutation** | **Delta G value** | **Effect on stability** |
| --- | --- | --- | --- |
| QQL14284.1 | R209I | -0.3569479 | Decrease |
| QQL14165.1 | S194L | -0.67079386 | Decrease |
| QQH16682.1 | G204R | -0.68136619 | Decrease |
| QQH15938.1 | R203K | -1.0587625 | Decrease |
| QQH17450.1 | S188P | -1.2061166 | Decrease |
| QQH16058.1 | R1951 | -0.6286178 | Decrease |
| QQ16646.1 | S202N | -0.67746599 | Decrease |
| QPB18048.1 | T2051 | -0.24030545 | Decrease |
| QQH16970.1 | Q289H | -0.39194072 | Decrease |
| QQH17450.1 | S327L | 0.55558303 | Increase |
| QQL14308.1 | T379I | -0.3859215 | Decrease |
| QQH15866.1 | D348Y | -0.99177863 | Decrease |
| QQH16526.1 | K374T | -1.1104731 | Decrease |
| QQH17294.1 | D377Y | -0.42988461 | Decrease |
| QQH16634.1 | S413I | -0.29679539 | Decrease |
| QQL14047.1 | D402Y | -0.59573415 | Decrease |
| QQH16058.1 | R14H | -0.81985283 | Decrease |
| QQH16034.1 | P13L | -0.79830902 | Decrease |
| QQH16778.1 | D03Y | -0.63221612 | Decrease |

**Predictions of missense SNPS by Provean:**

The cut off value is -2.5. if the value is equal to or below -2.5 the mutation is considered to be deleterious but if variation is above -2.5 then mutation is considered to be normal.

Table S6. Prediction analysis with the help of PROVEAN with the cut-off score of 2.5.

| **Accession no** | **Mutation** | **Provean score** | **Prediction** |
| --- | --- | --- | --- |
| QQL14284.1 | R209I | -2.455 | N |
| QQL14165.1 | S194L | -4.272 | D |
| QQH16682.1 | G204R | -1.656 | N |
| QQH15938.1 | R203K | -1.604 | N |
| QQH17450.1 | S188P | -2.918 | D |
| QQH16058.1 | R1951 | -3.993 | D |
| QQ16646.1 | S202N | -0.404 | N |
| QPB18048.1 | T2051 | -1.562 | N |
| QQH16970.1 | Q289H | -1.270 | N |
| QQH17450.1 | S327L | -3.022 | D |
| QQL14308.1 | T379I | -0.648 | N |
| QQH15866.1 | D348Y | -0.588 | N |
| QQH16526.1 | K374T | -0.819 | N |
| QQH17294.1 | D377Y | -1.779 | N |
| QQH16634.1 | S413I | 0.311 | N |
| QQL14047.1 | D402Y | -1.449 | N |
| QQH16058.1 | R14H | -0.609 | N |
| QQH16034.1 | P13L | -1.230 | N |
| QQH16778.1 | D03Y | -0.103 | N |

**Predictions of missense SNPS by POLYPHEN-2**

The score is between 0 and 1, if the score is near 1 then mutation is damaging but if this is close to 0 then it is benign.

Table S7. Prediction analysis of missense with the help of POLYPHEN-2.

| **Accession no** | **Mutation** | **POLYPHEN-2** | **Effect on stability** |
| --- | --- | --- | --- |
| QQL14284.1 | R209I | 0.998 | Probably Damaging |
| QQL14165.1 | S194L | 0.994 | Probably Damaging |
| QQH16682.1 | G204R | 1.000 | Probably Damaging |
| QQH15938.1 | R203K | 0.969 | Probably Damaging |
| QQH17450.1 | S188P | 0.994 | Probably Damaging |
| QQH16058.1 | R1951 | 0.101 | Benign |
| QQ16646.1 | S202N | 0.994 | Probably Damaging |
| QPB18048.1 | T2051 | 0.000 | Benign |
| QQH16970.1 | Q289H | 0.994 | Probably Damaging |
| QQH17450.1 | S327L | 0.000 | Benign |
| QQL14308.1 | T379I | 0.000 | Benign |
| QQH15866.1 | D348Y | 1.000 | Probably Damaging |
| QQH16526.1 | K374T | 0.997 | Probably Damaging |
| QQH17294.1 | D377Y | 1.000 | Probably Damaging |
| QQH16634.1 | S413I | 0.998 | Probably Damaging |
| QQL14047.1 | D402Y | 1.000 | Probably Damaging |
| QQH16058.1 | R14H | 0.998 | Probably Damaging |
| QQH16034.1 | P13L | 1.000 | Probably Damaging |
| QQH16778.1 | D03Y | 1.000 | Probably Damaging |

**DOCKTHOR:**

Table S8. Energy differences for berberine wild type and mutated nucleocapsid protein of SARS COV-2.

| **Mutation** | **Affinity** | **Total energy** | **Vdw energy** | **Elec. energy** |
| --- | --- | --- | --- | --- |
| Normal | -8.190 | 60.542 | -19.904 | 1.191 |
| R209I | -8.191 | 60.331 | -19.907 | 0.985 |
| S194L | -8.191 | 60.539 | -19.904 | 1.189 |
| S188P | -8.192 | 60.542 | -19.904 | 1.193 |
| R195I | -8.190 | 60.395 | -19.904 | 1.044 |
| S202N | -8.191 | 60.228 | -19.910 | 0.885 |
| T205I | -8.191 | 60.537 | -19.909 | 1.192 |
| S327L | -8.190 | 60.542 | -19.905 | 1.194 |
| T379I | -8.192 | 60.541 | -19.906 | 1.194 |
| S413I | -8.192 | 60.541 | -19.911 | 1.198 |
| R14H | -8.191 | 60.470 | -19.901 | 1.117 |
| P13L | -8.210 | 60.451 | -19.898 | 1.134 |
| Q289H | -8.143 | 60.532 | -19.905 | 1.141 |
| R203K | -8.176 | 60.702 | -19.923 | 1.172 |
| G204R | -8.178 | 60.243 | -19.928 | 1.142 |
| D348Y | -8.156 | 60.743 | -19.923 | 1.171 |
| K374T | -8.176 | 60.298 | -19.907 | 1.148 |
| D377Y | -8.139 | 60.489 | -19.912 | 1.138 |
| D402Y | -8.172 | 60.523 | -19.920 | 1.142 |
| D03Y | -8.141 | 60.743 | -19.902 | 1.132 |

Table S9. Energy differences for DISOGEN between wild type and mutated nucleocapsid protein of SARS COV-2.

| **Mutation** | **Affinity** | **Total energy** | **Vdw energy** | **Elec. energy** |
| --- | --- | --- | --- | --- |
| Normal | -8.218 | 37.293 | -19.972 | -4.093 |
| R209I | -8.220 | 37.295 | -19.879 | -4.190 |
| S194L | -8.215 | 37.294 | -20.044 | -4.011 |
| S188P | -8.220 | 37.295 | -19.917 | -4.146 |
| R195I | -8.214 | 37.292 | -20.040 | -4.020 |
| S202N | -8.215 | 37.289 | -20.037 | -4.025 |
| T205I | -8.215 | 37.293 | -20.046 | -4.016 |
| S327L | -8.216 | 37.292 | -20.024 | -4.044 |
| T379I | -8.213 | 37.294 | -20.049 | -4.010 |
| S413I | -8.213 | 37.294 | -20.051 | -4.009 |
| R14H | -8.216 | 37.294 | -20.007 | -4.059 |
| P13L | -8.132 | 37.289 | -20.014 | -4.043 |
| Q289H | -8.243 | 37.297 | -20.032 | -4.045 |
| R203K | -8.312 | 37.292 | -20.051 | -4.013 |
| G204R | -8.218 | 37.294 | -20.032 | -4.065 |
| D348Y | -8.210 | 37.284 | -20.028 | -4.022 |
| K374T | -8.206 | 37.292 | -20.018 | -4.051 |
| D377Y | -8.214 | 37.293 | -20.059 | -4.022 |
| D402Y | -8.208 | 37.294 | -20.023 | -4.043 |
| D03Y | -8.217 | 37.291 | -20.054 | -4.032 |

Table S10. Energy differences for CURCUMIN between wild type and mutated nucleocapsid protein of SARS COV-2.

| **Mutation** | **Affinity** | **Total energy** | **Vdw energy** | **Elec. energy** |
| --- | --- | --- | --- | --- |
| Normal | -7.674 | 37.249 | -15.257 | -14.499 |
| R209I | -7.789 | 38.696 | -13.630 | -13.338 |
| S194L | -7.927 | 37.601 | -19.791 | -9.918 |
| S188P | -7.587 | 36.985 | -17.385 | -12.509 |
| R195I | -7.584 | 36.880 | -16.557 | -13.412 |
| S202N | -7.594 | 36.827 | -16.511 | -13.663 |
| T205I | -7.703 | 38.252 | -18.870 | -10.099 |
| S327L | -7.571 | 37.397 | -15.598 | -14.663 |
| T379I | -7.594 | 36.817 | -16.557 | -13.527 |
| S413I | -7.567 | 37.586 | -15.385 | -14.631 |
| R14H | -7.572 | 37.199 | -15.836 | -14.619 |
| P13L | -7.684 | 37.001 | -15.254 | -14.332 |
| Q289H | -7.688 | 36.993 | -15.369 | -10.982 |
| R203K | -7.599 | 37.908 | -15.234 | -13.787 |
| G204R | -7.192 | 37.901 | -19.001 | -13.910 |
| D348Y | -7.905 | 36.913 | -14.990 | -12.034 |
| K374T | -7.130 | 37.009 | -16.980 | -14.098 |
| D377Y | -7.562 | 36.888 | -15.421 | -15.001 |
| D402Y | -7.192 | 37.092 | -13.098 | -14.765 |
| D03Y | -7.987 | 36.678 | -12.987 | -13.098 |

Table S11. Energy differences for Apigenin between wild type and mutated nucleocapsid protein of SARS COV-2.

| **Mutation** | **Affinity** | **Total energy** | **Vdw energy** | **Elec. energy** |
| --- | --- | --- | --- | --- |
| Normal | -7.184 | 14.416 | 10.022 | 15.579 |
| R209I | -7.531 | 14.883 | -11.924 | -12.590 |
| S194L | -7.343 | 12.222 | -11.117 | -15.953 |
| S188P | -7.538 | 14.911 | -12.447 | -12.141 |
| R195I | -7.393 | 13.566 | -11.288 | -14.431 |
| S202N | -7.537 | 14.922 | -11.120 | -13.349 |
| T205I | -7.217 | 12.287 | -12.139 | -14.498 |
| S327L | -7.152 | 14.497 | -9.480 | -14.983 |
| T379I | -7.172 | 13.514 | -10.397 | -14.943 |
| S413I | -7.328 | 12.057 | -12.193 | -14.695 |
| R14H | -7.233 | 14.143 | -10.952 | -13.862 |
| P13L | -7.342 | 12.452 | -9.875 | -14.594 |
| Q289H | -7.986 | 13.656 | -10.475 | -14.509 |
| R203K | -7.845 | 14.987 | -12.004 | -15.298 |
| G204R | -7.134 | 14.314 | -9.043 | -13.277 |
| D348Y | -7.685 | 12.482 | -12.908 | -15.324 |
| K374T | -7.523 | 13.991 | -11.879 | -13.009 |
| D377Y | -7.034 | 12.923 | -9.237 | -14.001 |
| D402Y | -7.976 | 14.078 | -9.576 | -15.301 |
| D03Y | -7.623 | 13.065 | -10.901 | -12.980 |

Table S12. Energy differences for EMODIN between wild type and mutated nucleocapsid protein of SARS-COV-2.

| **Mutation** | **Affinity** | **Total energy** | **Vdw energy** | **Elec. energy** |
| --- | --- | --- | --- | --- |
| Normal | -7.529 | 19.246 | -14.183 | -8.863 |
| R209I | -7.531 | 19.258 | -14.237 | -8.791 |
| S194L | -7.876 | 18.773 | -16.377 | -6.934 |
| S188P | -7.518 | 19.280 | -14.254 | -8.749 |
| R195I | -7.539 | 19.253 | -14.172 | -8.845 |
| S202N | -7.537 | 19.256 | -14.156 | -8.866 |
| T205I | -7.524 | 19.250 | -14.211 | -8.820 |
| S327L | -7.528 | 19.254 | -14.184 | -8.839 |
| T379I | -7.537 | 19.246 | -14.183 | -8.850 |
| S413I | -7.533 | 19.252 | -14.242 | -8.788 |
| R14H | -7.538 | 19.254 | -14.191 | -8.841 |
| P13L | -7.540 | 19.259 | -14.146 | -8.871 |
| Q289H | -7.808 | 18.987 | -14.234 | -8.654 |
| R203K | -7.519 | 19.358 | -14.153 | -8.753 |
| G204R | -7.891 | 19.258 | -14.312 | -8.692 |
| D348Y | -7.543 | 18.976 | -14.536 | -8.611 |
| K374T | -7.578 | 19.207 | -14.296 | -8.917 |
| D377Y | -7.599 | 19.396 | -16.300 | -8.889 |
| D402Y | -7.521 | 19.281 | -14.309 | -8.754 |
| D03Y | -7.532 | 19.230 | -16.459 | -8.443 |

**References**

1. Chatterjee B, Thakur SS. SARS-CoV-2 Infection triggers phosphorylation: potential target for Anti-COVID-19 therapeutics. *Front. Immunol*. 13, 1-14 (2022).
2. Bouhaddou M, Memon D, Meyer B, *et al.* The global phosphorylation landscape of SARS-CoV-2 infection. *Cell*. 180, 685–712 (2020).
3. Szklarczyk D, Gable AL, Lyon D, *et al. STRING* v11: protein–protein association networks with increased coverage, supporting functional discovery in genome-wide experimental datasets. *Nucleic Acids Res*. 47, D607–D613 (2019).
4. Davidson AD, Williamson MK, Lewis S, *et al.* Characterisation of the transcriptome and proteome of SARS-CoV-2 reveals a cell passage induced in-frame deletion of the furin-like cleavage site from the spike glycoprotein. *Genome Med*. 12, 1-15 (2020).
5. Klann K, Bojkova D, Tascher G, Growth factor receptor signaling inhibition prevents SARS-CoV-2 replication. *Mol. Cell*. *80*, 164–174 (2020).
6. Hekman RM, Hume AJ, Goel RK, *et al.* Actionable cytopathogenic host responses of human alveolar type 2 cells to SARS-CoV-2. *Mol. Cell.* 80, 1104–1122 (2020).
7. Alavizadeh SH, Doagooyan M, Zahedipour F, *et al*. Antisense technology as a potential strategy for the treatment of coronaviruses infection: With focus on COVID-19. *IET Nanobiotechnol*. 16(3), 67-77(2022).
8. Luttens A, Gullberg H, Abdurakhmanov E, *et al.* Ultralarge Virtual Screening Identifies SARS-CoV-2 Main Protease Inhibitors with Broad-Spectrum Activity against Coronaviruses, *J. Am. Chem. Soc*. 144, 2905–2920 (2022).
9. Clyde A, Galanie S, Kneller DW, *et al.* High-Throughput Virtual Screening and Validation of a SARS-CoV-2 Main Protease Noncovalent Inhibitor, *J. Chem. Inf. Model*. *62*, 116–128 (2022).
10. Chiu W, Verschueren L, Van den Eynde C, *et al.* Development and optimization of a high-throughput screening assay for in vitro anti-SARS-CoV-2 activity: Evaluation of 5676 Phase 1 Passed Structures. *J. Med. Virol*. 94, 3101–3111 (2022).
11. C. Gorgulla C, Das KMP, Leigh KE *et al.* A multi-pronged approach targeting SARS-CoV-2 proteins using ultra-large virtual screening. *iScience*. **2021**, 24 (2), 102021.
12. Yamamoto KZ, Yasuo N, Sekijima M. Screening for inhibitors of main protease in SARS-CoV-2: *In Silico* and *In Vitro* approach avoiding peptidyl secondary amides, *J. Chem. Inf. Model*. 62, 350–358 (2022).
13. Pohler A, Abdelfatah S, Riedl M, *et al.* Potential coronaviral inhibitors of the nucleocapsid protein identified *in silico* and *in vitro* from a large natural product library. *Pharmaceuticals*. 15(9), 1046 (2022).
14. Yadav R, Imran M, Dhamija P, Suchal K, Handu S. Virtual screening and dynamics of potential inhibitors targeting RNA binding domain of nucleocapsid phosphoprotein from SARS-CoV-2, J. *Biomol. Struct. Dyn.* 39, 4433–4448 (2021).
15. Li Z, Li X, Huang Y-Y. *et al.* Identify potent SARS-CoV-2 main protease inhibitors via accelerated free energy perturbation-based virtual screening of existing drugs, *Proc. Natl. Acad. Sci.*117, 27381–27387 (2020).
16. MorselliGysi D , do Valle Í, Zitnik M, *et al*. Network medicine framework for identifying drug-repurposing opportunities for COVID-19, *Proc. Natl. Acad. Sci*. 118, e2025581118 (2021).
17. Ahmed F, Soomro AM, ChethikkattuveliSalih AR, *et al.* A comprehensive review of artificial intelligence and network based approaches to drug repurposing in Covid-19. *Biomed. Pharmacother*. 153, 113350 (2022).
18. Hofmarcher M, Mayr A, Rumetshofer E, *et al.* Large-scale ligand-based virtual screening for SARS-CoV-2 inhibitors using deep neural networks, *SSRN Electron. J.*1-7 (2020).
19. Pillaiyar T, Laufer S. Kinases as potential therapeutic targets for anti-coronaviral therapy, *J. Med. Chem*. 65, 955–982 (2022).
20. Liu X, Verma A, Garcia G, *et al*. Targeting the coronavirus nucleocapsid protein through GSK-3 inhibition. *Proc. Natl. Acad. Sci*. 118, e2113401118 (2021).
21. Gordon DE, Jang GM, Bouhaddou M, *et al*. A SARS-CoV-2 protein interaction map reveals targets for drug repurposing. *Nature*. 583, 459–468 (2020).
22. Licheva M, Raman B, Kraft C, Reggiori F. Phosphoregulation of the autophagy machinery by kinases and phosphatases. *Autophagy*.18 (1), 104-123 (2022).
23. Cheng C, Qi RZ, Paudel H, Zhu J. Regulation and function of protein kinases and phosphatases. *Enzyme Re*s 79408.9 (2011).
24. Deng K, Liu L, Tan X, *et al*. WIP1 promotes cancer stem cell properties by inhibiting p38 MAPK in NSCLC. *Signal. Transduct. Target Ther*. *5*, 36 (2020).
25. Theivendren P, Kunjiappan S, Yashoda MY, *et al.* Importance of protein kinase and its inhibitor: A Review. In: *Protein kinases.* Singh RK (Ed) (2021).
